# Supplementary material for: Gene refashioning through innovative shifting of reading frames in mosses
Source: Nat Commun. 2018 Apr 19;9:1555. doi: 10.1038/s41467-018-04025-x (PMC5908804; doi:10.1038/s41467-018-04025-x)
Supplement: Supplementary file 1 — Supplementary Information [file 41467_2018_4025_MOESM1_ESM.pdf]

Supplementary information

for Guan et al.

Gene refashioning through innovative shifting of reading frames in mosses

a

Organism	Blast Name	Score	Number of Hits	Description
cellular organisms			1688	
. Eukaryota	eukaryotes		997	
. . Viridiplantae	green plants		10	
. . . Embryophyta	land plants		8	
. . . . Marchantia polymorpha subsp. ruderalis	liverworts	246	1	Marchantia polymorpha subsp. ruderalis hits
. . . . Selaginella moellendorffii	club-mosses	230	4	Selaginella moellendorffii hits
. . . . Physcomitrella patens	mosses	196	1	Physcomitrella patens hits
. . . . Quercus suber	eudicots	107	2	Quercus suber hits
. . . Monoraphidium neglectum	green algae	90.1	2	Monoraphidium neglectum hits
. . Sordaria macrospora k-hell	ascomycetes	157	2	Sordaria macrospora k-hell hits
. . Phellinus noxius	basidiomycetes	152	1	Phellinus noxius hits
. . Thielavia terrestris NRRL 8126	ascomycetes	150	2	Thielavia terrestris NRRL 8126 hits
. . Spizellomyces punctatus DAOM BR117	chytrids	148	6	Spizellomyces punctatus DAOM BR117 hits
. . Madurella mycetomatis	ascomycetes	147	2	Madurella mycetomatis hits
. . Hydnomerulius pinastri MD-312	basidiomycetes	146	2	Hydnomerulius pinastri MD-312 hits
. . Neurospora tetrasperma FGSC 2508	ascomycetes	146	2	Neurospora tetrasperma FGSC 2508 hits
. . Neurospora tetrasperma FGSC 2509	ascomycetes	146	1	Neurospora tetrasperma FGSC 2509 hits
. . Exophiala spinifera	ascomycetes	146	2	Exophiala spinifera hits
. . Neurospora crassa OR74A	ascomycetes	145	2	Neurospora crassa OR74A hits
. . Neurospora crassa	ascomycetes	145	1	Neurospora crassa hits
. . Rhizopogon vinicolor AM-OR11-026	basidiomycetes	144	1	Rhizopogon vinicolor AM-OR11-026 hits
. . Saitoella complicata NRRL Y-17804	ascomycetes	145	4	Saitoella complicata NRRL Y-17804 hits
. . Rhizopogon vesiculosus	basidiomycetes	144	1	Rhizopogon vesiculosus hits
. . Tulasnella calospora MUT 4182	basidiomycetes	144	2	Tulasnella calospora MUT 4182 hits
. . Ramazzottius varieornatus	tardigrades	144	3	Ramazzottius varieornatus hits
. . Chaetomium globosum CBS 148.51	ascomycetes	142	2	Chaetomium globosum CBS 148.51 hits
. . Pisolithus microcarpus 441	basidiomycetes	142	1	Pisolithus microcarpus 441 hits
. . Fomitiporia mediterranea MF3/22	basidiomycetes	142	2	Fomitiporia mediterranea MF3/22 hits
. . Kwoniella heveanensis BCC8398	basidiomycetes	141	1	Kwoniella heveanensis BCC8398 hits
. . Kwoniella heveanensis CBS 569	basidiomycetes	141	1	Kwoniella heveanensis CBS 569 hits
. . Paxillus involutus ATCC 200175	basidiomycetes	141	1	Paxillus involutus ATCC 200175 hits
[illegible]	[illegible]	[illegible]	[illegible]	

b

Organism	Blast Name	Score	Number of Hits	Description
cellular organisms			1727	
. Eukaryota	eukaryotes		718	
. . Viridiplantae	green plants		18	
. . . Chlorophyceae	green algae		11	
. . . . Monoraphidium neglectum	green algae	544	6	Monoraphidium neglectum hits
. . . . Gonium pectorale	green algae	118	1	Gonium pectorale hits
. . . . Tetrabaena socialis	green algae	114	1	Tetrabaena socialis hits
. . . . Chlamydomonas reinhardtii	green algae	110	1	Chlamydomonas reinhardtii hits
. . . . Volvox carteri f. nagariensis	green algae	107	2	Volvox carteri f. nagariensis hits
. . . Selaginella moellendorffii	club-mosses	93.1	4	Selaginella moellendorffii hits
. . . Quercus suber	eudicots	91.9	2	Quercus suber hits
. . . Physcomitrella patens	mosses	83.4	1	Physcomitrella patens hits
. . Colletotrichum chlorophyti	ascomycetes	120	1	Colletotrichum chlorophyti hits
. . Coniochaeta ligniaria NRRL 30616	ascomycetes	120	1	Coniochaeta ligniaria NRRL 30616 hits
. . Colletotrichum orbiculare MAFF 240422	ascomycetes	117	1	Colletotrichum orbiculare MAFF 240422 hits
. . Allomyces macrogynus ATCC 38327	blastocladiomycetes	116	2	Allomyces macrogynus ATCC 38327 hits
. . Trichoderma reesei QM6a	ascomycetes	115	4	Trichoderma reesei QM6a hits
. . Trichoderma reesei RUT C-30	ascomycetes	115	2	Trichoderma reesei RUT C-30 hits
. . Trichoderma parareesei	ascomycetes	114	2	Trichoderma parareesei hits
. . Coniosporium apollinis CBS 100218	ascomycetes	111	2	Coniosporium apollinis CBS 100218 hits
. . Podospora anserina S mat+	ascomycetes	110	9	Podospora anserina S mat+ hits
. . Hypoxylon sp. EC38	ascomycetes	110	3	Hypoxylon sp. EC38 hits
. . Pyronema omphalodes CBS 100304	ascomycetes	110	1	Pyronema omphalodes CBS 100304 hits
. . Fusarium pseudograminearum CS3096	ascomycetes	109	2	Fusarium pseudograminearum CS3096 hits
. . Fusarium pseudograminearum CS3220	ascomycetes	109	1	Fusarium pseudograminearum CS3220 hits
. . Fusarium pseudograminearum CS3427	ascomycetes	109	1	Fusarium pseudograminearum CS3427 hits
. . Fusarium pseudograminearum CS3487	ascomycetes	109	1	Fusarium pseudograminearum CS3487 hits
. . Elaphomyces granulatus	ascomycetes	113	1	Elaphomyces granulatus hits
. . Spizellomyces punctatus DAOM BR117	chytrids	108	6	Spizellomyces punctatus DAOM BR117 hits
. . Fusarium oxysporum f. sp. melonis 26406	ascomycetes	107	7	Fusarium oxysporum f. sp. melonis 26406 hits
. . Schizosaccharomyces pombe	ascomycetes	107	2	Schizosaccharomyces pombe hits
. . Schizosaccharomyces pombe 972h-	ascomycetes	107	1	Schizosaccharomyces pombe 972h- hits

Supplementary Figure 1. (a) Partial taxonomy distribution generated from BLAST search of the NCBI non-redundant (*nr*) protein sequence database using hemerythrin from *Marchantia polymorpha* (JGI ID: Mapoly0042s0056.1). Hits from only one green alga *Monoraphidium neglectum* were found. **(b)** Partial taxonomy distribution generated from BLAST search of nr database using the *Monoraphidium* sequence identified above, indicating all green algal hits were from Chlorophyceae. In both searches, E-value = 1e-8 was used as cutoff; land plant and algal hemerythrin sequences were more similar to fungal homologs than to each other.

a

hypothetical protein M407DRAFT_241621 [Tulasnella calospora MUT 4182]
Sequence ID: KIO31868.1 Length: 224 Number of Matches: 1

Range 1: 41 to 156 GenPept Graphics ▼ Next Match ▲ Pr

Score	Expect	Method	Identities	Positives	Gaps
95.4 bits(310)	2e-22	Compositional matrix adjust.	55/120(46%)	75/120(62%)	7/120(5%)

```
Query  2    IILEVKHDHAELEECFQRYKKAHSKGQDHEARNLFNQFVWEISRHSVSEELILYPMMDL-  60
            I  E+ +DH ELEE +  Y+ A S    + E      NQF WE++RHSV EEL+LYP  +
Sbjct  41   ISEEITQDHRELEEYYGNYRTATS---EKEKTQWANQFCWELARHSVGEELVLYPAFEKH  97

Query  61   LGDRGKELADQSREDHHRTKEILAELQT--ISDPSLFEKRLNIMMAELRDHMKMEEEEDL  118
            LG  GK++AD  R +H   K++L EL+   ++DP+ F   L  +M ELR+HMK EEE DL
Sbjct  98   LGPEGKQIADTDRAEHLEAKKLLYELEGTHVNDPN-FPTILKKLMEELREHMKSEEENDL  156
```

b

putative hemerythrin-like protein [Monoraphidium neglectum]
Sequence ID: XP_013901669.1 Length: 253 Number of Matches: 1
▶ See 1 more title(s)

Range 1: 75 to 192 GenPept Graphics ▼ Next Match ▲ F

Score	Expect	Method	Identities	Positives	Gaps
45.6 bits(140)	0.002	Compositional matrix adjust.	38/123(31%)	68/123(55%)	7/123(5%)

```
Query  1    DIILEVKHDHAELEECFQRYKKAHSKGQDHEAR-NLFNQFVWEISRHSVSEELILYPMMD  59
            +I+  +  DH  L   F     +   +G  H+ R +L +Q+V +++ HS+SEE +LYP
Sbjct  75   NIVDAITDDHKRL---FSYCDQLRGQGLGHDQREHLRHQLVHDLAVHSLSEEEVLYPA--  129

Query  60   LLGDRGKELADQSREDHHRTKEILAELQTI-SDPSLFEKRLNIMMAELRDHMKMEEEEDL  118
            L     G E+ ++   ++H   K IL ++   + +D   F   RL+ ++ ELR H++ EEE+ L
Sbjct  130  LKNACGNEMRNHVLDEHTTLKHILMDIDKMDADDPNFNPRLDELVHELRHHVQEEEEKVL  189

Query  119  AYL  121
              +
Sbjct  190  PMF  192
```

c

```
>ConsensusfromContig38372-abinit-gene-0.1
          Length = 182

 Score = 44.3 bits (103), Expect = 8e-06,   Method: Compositional matrix adjust.
 Identities = 34/115 (29%), Positives = 64/115 (55%), Gaps = 7/115 (6%)

Query: 6   VKHDHAELEECFQRYKKAHSKGQDHEARNLFNQFVWEISRHSVSEELILYPMMDLLGDRG 65
           ++ DH+ ++E + R+K    SK  + + R   NQF+ ++S+HSV+EE++ YP ++     G
Sbjct: 43  LQKDHSFIKEDYNRWKA--SKNAEEKIR-WANQFILDVSQHSVAEEIVYYPEIEKRQKGG 99

Query: 66  KELADQSREDHHRTKEILAELQTI--SDPSLFEKRLNIMMAELRDHMKMEEEEDL 118
                +  ++H   K +L  L  +  +DP  ++  L  + + L+ H+K EEE D
Sbjct: 100 SISKQKHLDEHQEVKNMLYNLDRMNYTDPG-YDALLERIYSALQ-HIKDEEENDF 152
```

Supplementary Figure 2. Land plant hemerythrins are more similar to fungal homologs than to those from chlorophyte green algae and glaucophytes. Figures show pairwise comparisons of the conserved HHE domain between liverwort *Marchantia polymorpha* (Mapoly0042s0093.1) and homologs from fungi, chlorophytes, and glaucophytes, respectively. Fungal and green algal sequences here are top hits from BLASTP search of nr database. **(a)** Sequence alignment between *Marchantia polymorpha* and the fungus *Tulasnella calospora*. **(b)** Sequence alignment between *Marchantia polymorpha* and chlorophyte *Monoraphidium neglectum*. **(c)** Sequence alignment between *Marchantia polymorpha* and the glaucophyte *Cyanophora paradoxa*.

a

>Partial sequence of the *Isoete yunguiensis* mosaic gene derived from fusion of oleosin and *Hr*

CCTCTCGCGATGCTGGGGACGAGCCTAACCATAGGAACCCTAATTCTGGTAATATCTTCGCCGCTGTTACTCCTTTTTAGCCCAATTCTAGTACC

TCTAGGGTTTGTGCTTTTCATGGCCGCGGCAGCCTTCGCGGCTATGGTGGCAGCAGGCAACGCAGTGGCATGGATCTACCGCTACAAAAAAGG

CCGCCACCCCATGGGATCCGATAAACTTGATGCAGCAATCCACATGTTCATACCCAGAAGAGATATCATCGACCTCGTTAGGGAGGATCATGC

TAAACTGGAAGAGGATTACGGTAATTACAAGAGCGCCAGCCGTAGAGGAGACCACTACGAGGCCAGAAAATGGTTCAACCAGTTTGTGTGGG

AAATTTCCAGACATTCTGTCTCCGAAGAGCTTGTTATGTATCCCCTCCTTGATGGGCTGGGCCCAAAAGGCCGAGATTTGGCATATCAATCCCG

TGCAGATCATCACAAGATCAAGGAATTGCTCACGGAATTGCAGCACAACACTGATTCTGAGGATTTTGATTCCAGAATGGAGACCATGATGTC

CAATCTCAGAGACCATATTAAGTTGGAAGAAAAGCGCGATGGAGATCTGGCTTGTTTGCGTGATAATATGGATCAGCAGGCTCGCGAGGCTGC

CGGAGCTACATTTGCACTGGGCAAGAATCT

>Translated peptide sequence of the *Isoete yunguiensis* mosaic gene

P L A M L G T S L T I G T L I L V I S S P L L L L F S P I L V P L G F V L F M A A A A F A A M V A A G N A V A W I Y R Y K K G R H P M G S D K

L D A A I H M F I P R R D I I D L V R E D H A K L E E D Y G N Y K S A S R R G D H Y E A R K W F N Q F V W E I S R H S V S E E L V M Y P L L

D G L G P K G R D L A Y Q S R A D H H K I K E L L T E L Q H N T D S E D F D S R M E T M M S N L R D H I K L E E K R D G D L A C L R D

N M D Q Q A R E A A G A T F A L G K N

b

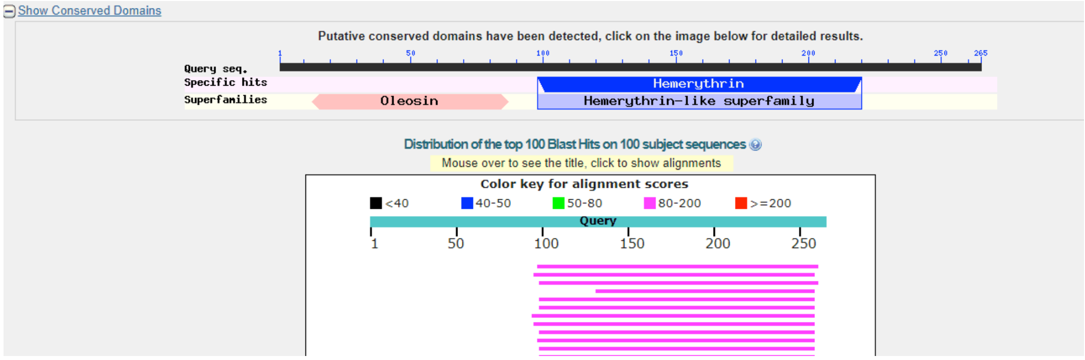

Supplementary Figure 3. (a) Partial nucleotide sequence and translated peptide sequence of the *oleosin* and *Hr* mosaic gene in the lycophyte *Isoetes yunguiensis* (GenBank accession number MG254883). **(b)** CDD comparison with *I. yunguiensis* protein sequence, indicating fusion of oleosin and HHE domain. Although the same gene fusion was also found in the liverwort *Treubia lacunosa* (1KP ID: FITN-2089742), its nucleotide sequence shared 100% identity with PYHZ-2006808, pointing to a possibility of contamination.

a

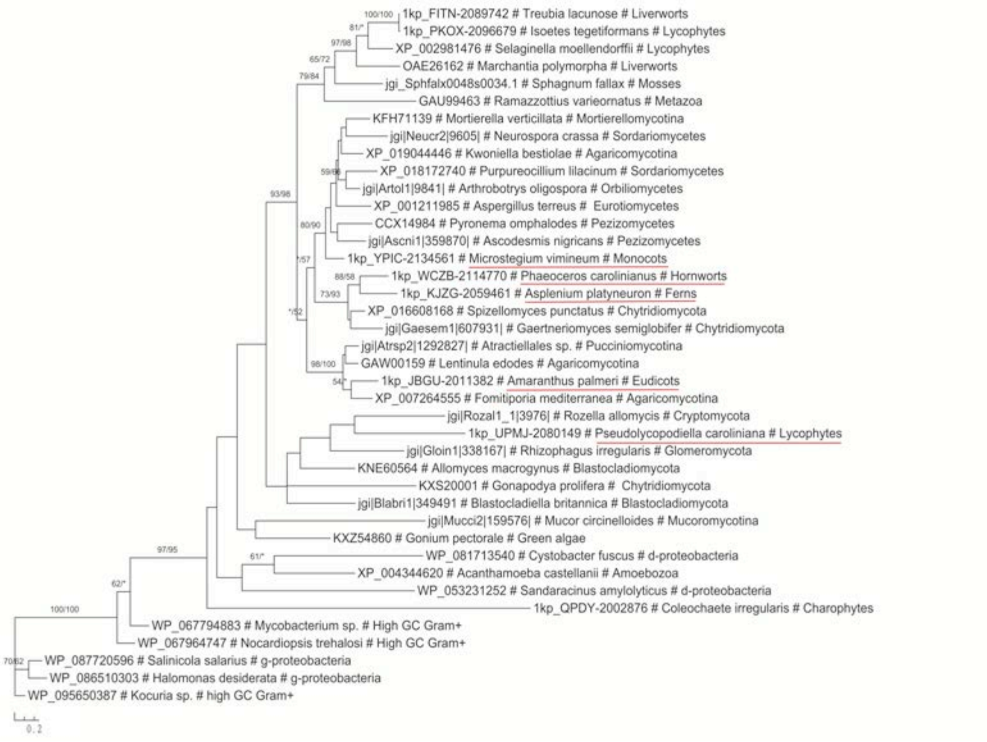

100/100
1kp_FITN-2089742 # Treubia lacunose # Liverworts
81/*
1kp_PKOX-2096679 # Isoetes tegetiformans # Lycophytes
97/98
XP_002981476 # Selaginella moellendorffii # Lycophytes
65/72
OAE26162 # Marchantia polymorpha # Liverworts
79/84
jgi_Sphfalx0048s0034.1 # Sphagnum fallax # Mosses
GAU99463 # Ramazzottius varieornatus # Metazoa
KFH71139 # Mortierella verticillata # Mortierellomycotina
jgi|Neucr2|9605| # Neurospora crassa # Sordariomycetes
XP_019044446 # Kwoniella bestiolae # Agaricomycotina
XP_018172740 # Purpureocillium lilacinum # Sordariomycetes
jgi|Artol1|9841| # Arthrobotrys oligospora # Orbiliomycetes
93/98
XP_001211985 # Aspergillus terreus # Eurotiomycetes
80/90
CCX14984 # Pyronema omphalodes # Pezizomycetes
jgi|Ascni1|359870| # Ascodesmis nigricans # Pezizomycetes
*/57
1kp_YPIC-2134561 # Microstegium vimineum # Monocots
88/58
1kp_WCZB-2114770 # Phaeoceros carolinianus # Hornworts
73/93
1kp_KJZG-2059461 # Asplenium platyneuron # Ferns
*/52
XP_016608168 # Spizellomyces punctatus # Chytridiomycota
jgi|Gaesem1|607931| # Gaertneriomyces semiglobifer # Chytridiomycota
jgi|Atrsp2|1292827| # Atractiellales sp. # Pucciniomycotina
98/100
GAW00159 # Lentinula edodes # Agaricomycotina
54/*
1kp_JBGU-2011382 # Amaranthus palmeri # Eudicots
XP_007264555 # Fomitiporia mediterranea # Agaricomycotina
jgi|Rozal1_1|3976| # Rozella allomycis # Cryptomycota
1kp_UPMJ-2080149 # Pseudolycopodiella caroliniana # Lycophytes
jgi|Gloin1|338167| # Rhizophagus irregularis # Glomeromycota
KNE60564 # Allomyces macrogynus # Blastocladiomycota
KXS20001 # Gonapodya prolifera # Chytridiomycota
jgi|Blabri1|349491 # Blastocladiella britannica # Blastocladiomycota
jgi|Mucci2|159576| # Mucor circinelloides # Mucoromycotina
KXZ54860 # Gonium pectorale # Green algae
97/95
61/*
WP_081713540 # Cystobacter fuscus # d-proteobacteria
XP_004344620 # Acanthamoeba castellanii # Amoebozoa
62/*
WP_053231252 # Sandaracinus amylolyticus # d-proteobacteria
100/100
1kp_QPDY-2002876 # Coleochaete irregularis # Charophytes
WP_067794883 # Mycobacterium sp. # High GC Gram+
WP_067964747 # Nocardiopsis trehalosi # High GC Gram+
70/62
WP_087720596 # Salinicola salarius # g-proteobacteria
WP_086510303 # Halomonas desiderata # g-proteobacteria
WP_095650387 # Kocuria sp. # high GC Gram+
0.2

b

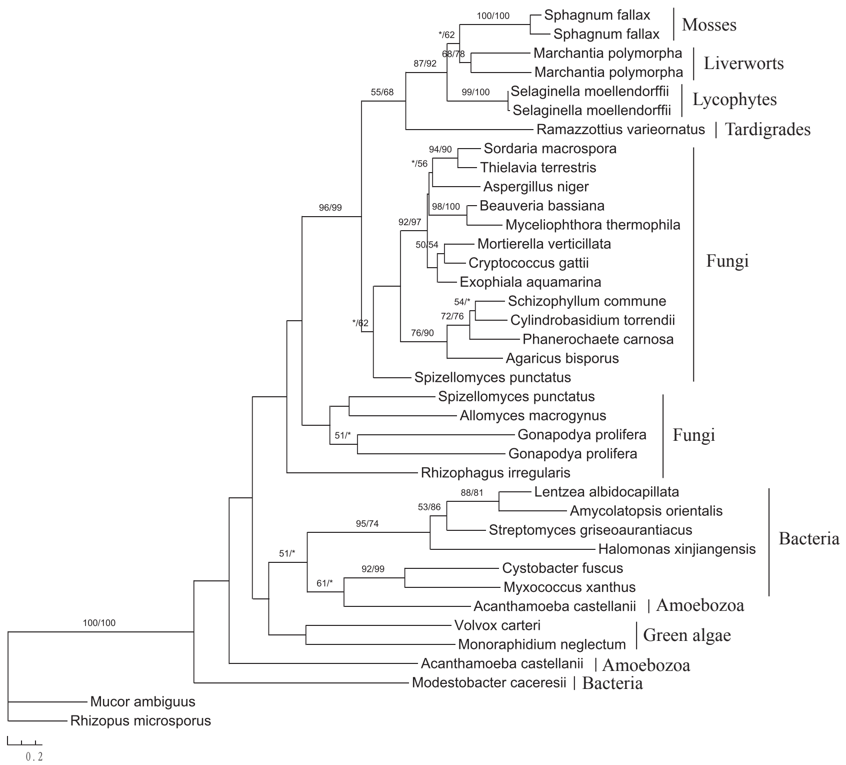

100/100
Sphagnum fallax
Sphagnum fallax
Mosses
*/62
68/78
Marchantia polymorpha
Marchantia polymorpha
Liverworts
87/92
99/100
Selaginella moellendorffii
Selaginella moellendorffii
Lycophytes
55/68
Ramazzottius varieornatus
Tardigrades
94/90
Sordaria macrospora
*/56
Thielavia terrestris
Aspergillus niger
96/99
98/100
Beauveria bassiana
Myceliophthora thermophila
92/97
50/54
Mortierella verticillata
Cryptococcus gattii
Exophiala aquamarina
Fungi
54/*
Schizophyllum commune
72/76
Cylindrobasidium torrendii
*/62
76/90
Phanerochaete carnosa
Agaricus bisporus
Spizellomyces punctatus
Spizellomyces punctatus
Allomyces macrogynus
51/*
Gonapodya prolifera
Gonapodya prolifera
Fungi
Rhizophagus irregularis
88/81
Lentzea albidocapillata
53/86
Amycolatopsis orientalis
95/74
Streptomyces griseoaurantiacus
Halomonas xinjiangensis
Bacteria
51/*
92/99
Cystobacter fuscus
61/*
Myxococcus xanthus
Acanthamoeba castellanii
Amoebozoa
Volvox carteri
Monoraphidium neglectum
Green algae
Acanthamoeba castellanii
Amoebozoa
Modestobacter caceresii
Bacteria
Mucor ambiguus
Rhizopus microsporus
0.2

Supplementary Figure 4. Molecular phylogeny of hemerythrin (Hr) protein sequences. LG and JTT substitution models were used in maximum and distance analyses, respectively, and four-gamma rate categories plus invariant sites were used in the analyses. Numbers above branches show bootstrap values from maximum likelihood and distance analyses, respectively. Asterisks indicate values lower than 50%. Nodes without values indicate that bootstrap support values from both analyses are lower than 50%. Taxonomic affiliations are after species names. **(a)** Phylogenetic analyses with a larger taxonomic sampling, including multiple angiosperm and seedless vascular plant sequences from 1KP (underlined in red). **(b)** Phylogenetic analyses with sequences of known scaffold information, mostly from genome sequence projects. The sequence of glaucophyte *Cyanophora paradoxa* was removed from the analyses because of its much shorter length. Inclusion of the *Cyanophora* sequence provided a similar topology, with much lower branch support values.

```
>Chr21: 12755680..12757359

12757359   cgcatgctcagcacctgtcgagcacacgcccgtaccagtcatcgtcctccccaatccacctttcgcctcctgcttcaatc

                                    M  Y  N  M  C  S  L  G  R  S  P  P  Y  I  N  Q  N  P  F  C  L
12757279   ttctctcatccacgtgcATGTATAATATGTGCAGCCTCGGTCGATCACCCCCGTATATCAATCAGAACCCGTTCTGCTTG

           C  T  L  S  D  L  Q  P  L  Y  H  S  L  S  V  I  L  Y  I  T  H  K  A  L  V  V  H
12757199   TGCACACTATCAGATTTGCAACCTCTCTATCACTCTCTTAGTGTAATTCTATATATCACTCACAAAGCTCTAGTTGTACA

            R  E  A  E  A  L  L  P  A  D  H  T  V  H  H  G  R  S  R  I  H  P  L  E  R  C  R
12757119   CCGTGAAGCCGAAGCACTTCTCCCAGCAGATCACACTGTCCACCATGGCCGCAGTCGCATACATCCCCTTGAGCGCTGTC
                                                       m  a  a  v  a  y  i  p  l  s  a  v

             F  R  E  V  G  N  H  P  S  Q  L  Q  Q  C  G  L  P  A  Q  R  G  H  C  C  V  Q
12757039   GCTTCCGCGAGGTTGGCAACCACCCAAGCCAGCTCCAGCAATGCGGCCTCCCAGCCCAGCGCGGGCATTGTTGCGTTCAA
           a  s  a  r  l  a  t  t  q  a  s  s  s  n  a  a  s  q  p  s  a  g  i  v  a  f  k

           A  S  C  H  A  I  L  P  A
12756959   GCGAGCTGTCACGCCATCTTGCCTGGTACTGAACCCGTCTCCTGGGGGCTTGTTCGTTGTGAGGATTGTCCCGCGCGGTC
            r  a  v  t  p  s  c  l

12756879   GATTGGATGGCATGTGAACAAGTGTAGTGCGATTGCTGAGCTCGGGAACCAAGTCCATGGCGAGCATGGGTGTGGTTCCT

12756799   GAGCTGAGCAGGAAGTGGGGACGTGAAATTTGGCCGCGGCCAGGGAGTGTGGGCTTGTTGCAATTGTGGGTGTTTGAGAG

                                                                             V  G  E  P  V  L  R
12756719   CGATGACAATGGATGTTTGGTGCTCTAACGTGAAAAACATGTTGTGTCTGGATCGCAGCAGTCGGCGAGCCAGTTCTGCG
                                                                              s  a  s  q  f  c  g

            R  P  R  H  F  L  Q  Q  Q  Q  Q  Q  Q  P  G  R  E
12756639   GCGTCCCCGTCACTTTCTCCAACAGCAGCAGCAGCAGCAGCCTGGTCGAGGTAAGTGCATCCATGTCTACCGCGCGTGTT
             v  p  v  t  f  s  n  s  s  s  s  s  s  l  v  e  v  s  a  s  m  s  t  a  r  v

12756559   GGGCAGTGTGCCTCCATGTCGATCGCAAGAGGTGGTGGTTTTGTTTTGGTCGACTTGCGCGCCGCAATCGTGACGTGGTG
           g  q  c  a  s  m  s  i  a  r  g  g  g  f  v  l  v  d  l  r  a  a  i  v  t  w  w

12756479   GATGAGTGTGGGTGGGAATTGCGCGGTTCTTCCAGCTGGGCACAGCTAATGCATTTTGGGGGGGGAGGGGGGGAGGGGGG
            m  s  v  g  g  n  c  a  v  l  p  a  g  h  s  *

                                                E  T  G  R  V  D  Q  M  L  R  H  S  E  E  F  G  G  G
12756399   GGGGGTGGGGGATGGGTTTGAGCAGAGGAAACAGGGCGGGTCGATCAGATGCTCCGCCACTCAGAAGAGTTTGGAGGTGG

            R  R  H  H  R  Q  S  E  A  G  P  Q  G  A  G  G  G  V  L  Q  L  Q  E  V  P  Q  A
12756319   GCGCCGACATCATCGACAAAGTGAAGCAGGACCACAAGGAGCTGGAGGAGGCGTACTCCAATTACAAGAAGTTCCACAAG

             G  Q  R  G  R  G  R  I  M  V  Q  P  V  R  V  G  D  L  S  P  C  R  H  R  G  A
12756239   CAGGGCAACGAGGAAGAGGCCGGATAATGGTTCAACCAGTTCGTGTGGGAGATCTCTCGCCATGCCGTCACCGAGGAGCT

                                       *

           G  V  V  P  V  D  R  V  A  G  R  Q  G  P  E  A  G  G  P  V  A  R  R  A  P  E  D
12756159   GGTGTTGTACCCGTTGATCGCGTCGCAGGGCGACAAGGGCCAGAAGCTGGCGGACCAGTCGCGCGACGAGCACCAGAAGA

            E  G  H  A  G  G  D  P  G  H  R  G  R  R  P  V  *
12756079   CGAAGGACATGCTGGTGGAGATCCAGGGCATCGAGGACGACGACCTGTTTGAGAAGAAGTTCGACGCCATCATGGACGTG

12755999   CTGCGAGAGCACGTGCAAAAGGAGGAGTCGGAGGATCTGGAGTACTTGAGGCAGAACGTGGACCAGGCAGCCCGCGAGGC

12755919   AGCGGGAACCACATTCGCGCTGGGCAAGAACATCGTGCCGACGAAGCCCCATGCGGCAGTGCCGAACAGGTCGGCGATAG

12755839   TGGAGGGCGCGCTGGGGCTGTTCGCGACTCCCTTGGACAAGCTGCGCGACGTGTTCAGGCCGTATCCCAAGGACAACTAG

12755759   aggagcaatttgtagctttttgtacatcagccccccgtcccgcgagtttgttgttgcaaataggtggctccgggatggat

          ATCG     YAN
          ATCG     AltYAN
          ATCG     Hr
```

Supplementary Figure 5. Sequences of *YAN*, *AltYAN*, and *Hr*. Black boxes indicate exons of *YAN*. Red letters show exons of *AltYAN*. Blue letters show the pseudogenized *Hr* region. Capital letters above the nucleotide sequences indicate the amino acids encoded by *YAN*, while lowercase letters below indicate those encoded by *AltYAN*.

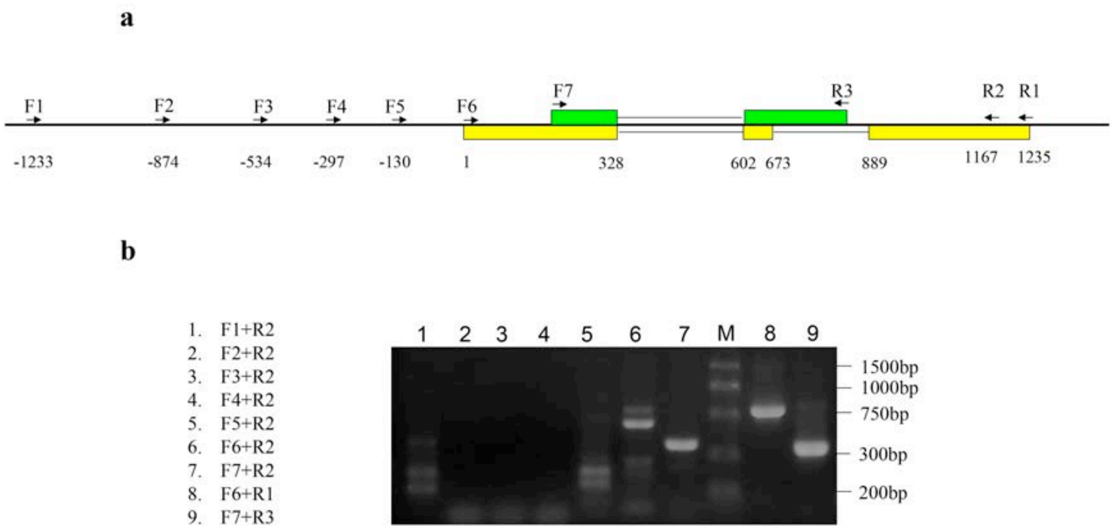

Supplementary Figure 6. RT-PCR confirmation of *YAN*, *AltYAN*, and *Hr*. (**a**) Schematic diagram of primers. To confirm the mRNA completeness of this locus, seven forward primers were designed, with F1-F5 annealing to the upstream and F6-F7 annealing to exons. Each forward primer cooperated with a reverse primer R2 for RT-PCR reactions. To further confirm the CDS sequences of *YAN* and *AltYAN*, two primer pairs, (F6, R1) and (F7, R3), were used to amplify the CDSs of *YAN* and *AltYAN*, respectively. Amplified CDSs were then sequenced. (**b**) Gel result of RT-PCR. F1-F5 cooperating with R2 failed to amplify the appropriate product, but F6 and F7 cooperating with R2 could amplify the appropriate product. The lengths of *YAN* and *AltYAN* CDS sequences were correct.

a

>*Physcomitrium sp.* *YAN* and *Hr* annotation

```
             M Y N M C S L G R S P P Y I N Q N S F C L
CTCATCCACGTGCATGTATAATATGTGCAGCCTCGGTCGATCACCCCCGTACATCAACCAGAACTCGTTCTGCTT
 C T L S D L Q P L Y H S L S V I L Y I T H K A L V
GTGCACACTATCAGATTTGCAACCTCTCTATCACTCGCTCAGTGTAATTCTATATATCACTCACAAAGCTCTAGT
 V H R E A E A L L P A D H T V H H G R S R I H P
TGTACACCGTGAAGCCGAAGCACTTCTCCCAGCAGATCACACTGTCCACCATGGCCGCAGTCGCATACATCC
                                                  m a a v a y i
 L E R C R S R E V G N H P S Q L Q Q C G L P A Q
CCTTGAGCGCTGTCGCTCCCGCGAGGTTGGCAACCACCCAAGCCAGCTCCAGCAATGCGGCCTCCCAGCCCA
p l s a v a p a r l a t t q a s s s n a a s q p
 C G H C C V Q A S C H A I L P
GTGCGGGCATTGTTGCGTTCAAGCGAGCTGCCACGCCATCTTGCCTG
s a g i v a f k r a a t p s c l

A V G E P V L R R P R H F L Q Q Q Q Q Q Q P G R
CAGTCGGCGAGCCAGTTCTGCGGCGTCCCCGTCACTTTCTCCAACAGCAGCAGCAGCAGCAGCCTGGTCGAG
 q s a s q f c g v p v t f s n s s s s s s l v e

E E T G R V D Q M L R H S E E F G G G R R H H R
AGGAAACAGGGCGGGTCGATCAGATGCTCCGCCACTCAGAAGAGTTTGGAGGTGGGCGCCGACATCATCGAC
 r k q g g s i r c s a t q k s l e v g a d i i d
Q S E A G P Q G A G G G V L Q L Q E V P Q A G Q
AAAGTGAAGCAGGACCACAAGGAGCTGGAGGAGGCGTACTCCAATTACAAGAAGTTCCACAAGCAGGGCAA
 k v k q d h k e l e e a y s n y k k f h k q g n
 R G R G R K M V Q P V R V G D L A P C R H R G A
CGAGGAAGAGGCCGGAAAATGGTTCAACCAGTTCGTGTGGGAGATCTCGCGCCATGCCGTCACCGAGGAGCT
 e e e a g k w f n q f v w e i s r h a v t e e l
 G V V P V D R V A G R Q G P E A G G P V A R R A
GGTGTTGTACCCGTTGATCGCGTCGCAGGGCGACAAGGGCCAGAAGCTGGCGGACCAGTCGCGCGACGAGC
 v l y p l i a s q g d k g q k l a d q s r d e
 P E D E G H A G G D P G H R G R R P V *
ACCAGAAGACGAAGGACATGCTGGTGGAGATCCAGGGCATCGAGGACGACGACCTGTTTGAGAAGAAGTTC
 h q k t k d m l v e i q g i e d d d l f e k k f
GACGCAATCATGGACGTGCTGCGAGAGCACGTGCAAAAGGAGGAGTCGGAGGATCTGGAGTACTTGAGGCA
 d a i m d v l r e h v q k e e s e d l e y l r q
GAACGTGGAACAGGCAGCCCGCGAGGCAGCGGGAACCACATTCGCGCTGGGCAAGAACATCGTGCCGACGA
 n v e q a a r e a a g t t f a l g k n i v p t
AGCCCCATGCGGCAGTGCCGAACAGGTCGGCGATAGTGGAGGGCGCCCTGGGGCTGTTCGCGACTCCCTTGG
k p h a a v p n r s a i v e g a l g l f a t p l
ACAAGCTGCGCGACGTGTTCACGCCGTATCCCAAGGACAACTAGAGGAGCAATTTGTAGCTTTTTGTACATCA
d k l r d v f t p y p k d n *
```

ATCG (boxed) *YAN*

ATCG (blue) *Hr*

b

```
                         1       10        20        30        40        50        60        70        80        90        100       110       120       130
                         |--------+---------+---------+---------+---------+---------+---------+---------+---------+---------+---------+---------+---------|
Physcomitrium_sp_for     MAAVAYIPLSAVAPARLATTQASSSNAASQPSAGIVAFKRAATPSCLQSASQFCGVPVTFSNSSSSSSLVERKQGGSIRCSATQKSLEVGADIIDKVKQDHKELEEAYSNYKKFHKQGNEEEAGKWFNQF
Sphfalx0048s0034.1                                                                               MSSHVTTTHHVPGDLMDEIKHDHREFEEFYTNYKRAYAAGDEKEAEKWFNQL
Consensus                ..............................................................................rcSatqksheVgaDiiDe!KqDHrElEEaYsNYKrahaaG#EeEAeKWFNQl

                         131     140       150       160       170       180       190       200       210       220       230       240       250       260
                         |--------+---------+---------+---------+---------+---------+---------+---------+---------+---------+---------+---------+---------|
Physcomitrium_sp_for     VWEISRHAVTEELVLYPLIASQGDKGQKLADQSRDEHQKTKDMLVEIQGIEDDDLFEKKFDAIMDVLREHVQKEESEDLEYLRQNVEQAAREAAGTTFALGKNIVPTKPHAAVPNRSAIVEGALGLFATP
Sphfalx0048s0034.1       VWELSRHMVGEELVVYPLLDAMGPEGKEMADKDRADHQKVKKELEEIHRLSDPGLFEATLDRIMQDLRQHTNKEETQDLEYLKQHADKKSLENAAKAFKYGKKMAPTRPHPGIPNRSAALEAALAFFVTP
Consensus                VWEiSRHaVgEELVlYPLiaaqGdeGqe$ADqdRa#HQKtKdeLeEIqrieDddLFEaklDaIM#dLR#Ht#KEEs#DLEYLrQna#qaarEaAakaFalGKniaPTrPHaa!PNRSAalEaALalFaTP

                         261     270       280     289
                         |--------+---------+--------|
Physcomitrium_sp_for     LDKLRDVFTPYPKDN
Sphfalx0048s0034.1       LDKLRDMFTPFPKEEHVPYQPKQEEHAKK
Consensus                LDKLRDmFTP%PK##..............
```

c

```
                         1       10        20        30        40        50        60        70        80        90        100       110       120       130
                         |--------+---------+---------+---------+---------+---------+---------+---------+---------+---------+---------+---------+---------|
Physcomitrium_sp         MYNMCSLGRSPPYINQNSFCLCTLSDLQPLYHSLSVILYITHKALVVHREAEALLPADHTVHHGRSRIHPLERCRSREVGNHPSQLQQCGLPAQCGHCCVQASCHAILPAVGEPVLRRPRHFLQQQQQQQ
Pp3c21_19720V3.2         MYNMCSLGRSPPYINQNPFCLCTLSDLQPLYHSLSVILYITHKALVVHREAEALLPADHTVHHGRSRIHPLERCRFREVGNHPSQLQQCGLPAQRGHCCVQASCHAILPAVGEPVLRRPRHFLQQQQQQQ
Consensus                MYNMCSLGRSPPYINQNpFCLCTLSDLQPLYHSLSVILYITHKALVVHREAEALLPADHTVHHGRSRIHPLERCRfREVGNHPSQLQQCGLPAQrGHCCVQASCHAILPAVGEPVLRRPRHFLQQQQQQQ

                         131     140       150       160       170       180       190       200       210       220       230       240     248
                         |--------+---------+---------+---------+---------+---------+---------+---------+---------+---------+---------+-------|
Physcomitrium_sp         PGREETGRVDQMLRHSEEFGGGRRHHRQSEAGPQGAGGGVLQLQEVPQAGQRGRGRKMVQPVRVGDLAPCRHRGAGVVPVDRVAGRQGPEAGGPVARRAPEDEGHAGGDPGHRGRRPV
Pp3c21_19720V3.2         PGREETGRVDQMLRHSEEFGGGRRHHRQSEAGPQGAGGGVLQLQEVPQAGQRGRGRIMVQPVRVGDLSPCRHRGAGVVPVDRVAGRQGPEAGGPVARRAPEDEGHAGGDPGHRGRRPV
Consensus                PGREETGRVDQMLRHSEEFGGGRRHHRQSEAGPQGAGGGVLQLQEVPQAGQRGRGRiMVQPVRVGDLaPCRHRGAGVVPVDRVAGRQGPEAGGPVARRAPEDEGHAGGDPGHRGRRPV
```

Supplementary Figure 7. **(a)** Sequence and annotation of RT-PCR product of the HHE region in *Physcomitrium sp*. (GenBank accession number MG254884). Living materials were collected from Xinping, Yunnan, China. Amino acids encoded by the *Hr* gene are shown by lower case characters below the nucleotide sequence. Upper case characters above the nucleotide sequence indicate amino acids encoded by *YAN*. Boxes show exons of *YAN* (GenBank accession number MG254882). **(b)** Alignment of putative Hr protein sequences from *Physcomitrium sp*. and *Sphagnum fallax*. **(c)** Alignment of putative YAN protein sequences from *Physcomitrium sp*. and *Physcomitrella patens*.

a

>*Physcomitrium sp. AltYAN* annotation

```
ATGGCCGCAGTCGCATACATCCCCTTGAGCGCTGTCGCTCCCGCGAGGTTG
 M   A   A   V   A   Y   I   P   L   S   A   V   A   P   A   R   L
GCAACCACCCAAGCCAGCTCCAGCAATGCGGCCTCCCAGCCCAGTGCGGGC
 A   T   T   Q   A   S   S   S   N   A   A   S   Q   P   S   A   G
ATTGTTGCGTTCAAGCGAGCTGCCACGCCATCTTGCCTG
 I   V   A   F   K   R   A   A   T   P   S   C   L

TCGGCGAGCCAGTTCTGCGGCGTCCCCGTCACTTTCTCCAACAGCAGCAGC
 S   A   S   Q   F   C   G   V   P   V   T   F   S   N   S   S   S
GGCAGCAGCCTGGTCGAGGTAAGTGCATCCATGTCCACCGCGTGTGCTGGG
 G   S   S   L   V   E   V   S   A   S   M   S   T   A   C   A   G
CAGTGTGCCTCCATGTCGATCGCCAGAGGTGGTGGTTTTGTTTTGGTCGAC
 Q   C   A   S   M   S   I   A   R   G   G   G   F   V   L   V   D
TTGCGCGCCGCAATCGTGACGTGGTGGATGA……
 L   R   A   A   I   V   T   W   W   M
```

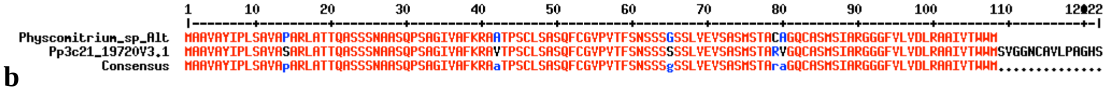

b

Supplementary Figure 8. **(a)** Partial sequence and annotation of RT-PCR product of *AltYAN* in *Physcomitrium sp*. (GenBank accession number: MG254881). Primers specific to the *AltYAN* region were used for RT-PCR reactions. Living materials were collected from Xinping, Yunnan, China. Amino acids encoded by *AltYAN* are shown below the nucleotide sequence. **(b)** Alignment of putative AltYAN protein sequences from *Physcomitrium sp*. and *Physcomitrella patens*.

a

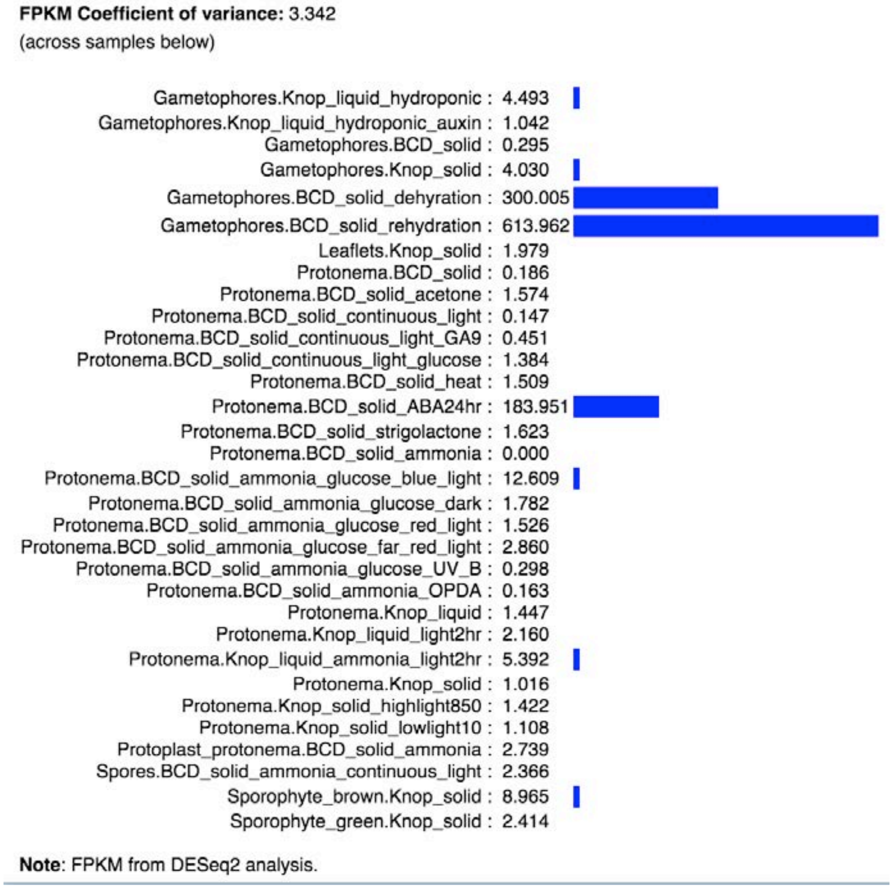

b

Gene models – Pp3c21_19720

Gene models

Transcripts: 2 Exons: 5 Introns: 3 CDSs: 2

Expression

Pearson Correlated Expression

There are 3 genes with correlated expression > 0.85

Gene	Define	Correlation
Pp3c16_15390	(1 of 4) PTHR24096:SF219 - LONG CHAIN ACYL-COA SYNTHETASE 8	0.8926
Pp3c19_19049		0.86176
Pp3c8_4710		0.85877

Supplementary Figure 9. Transcription data of *YAN/AltYAN* from Phytozome (https://phytozome.jgi.doe.gov/phytomine/report.do?id=237468538). **(a)** *YAN/AltYAN* is strongly induced by both dehydration and rehydration. **(b)** *YAN/AltYAN* is co-expressed with multiple other genes, including long-chain acyl-CoA synthetase 8 (*LACS 8*).

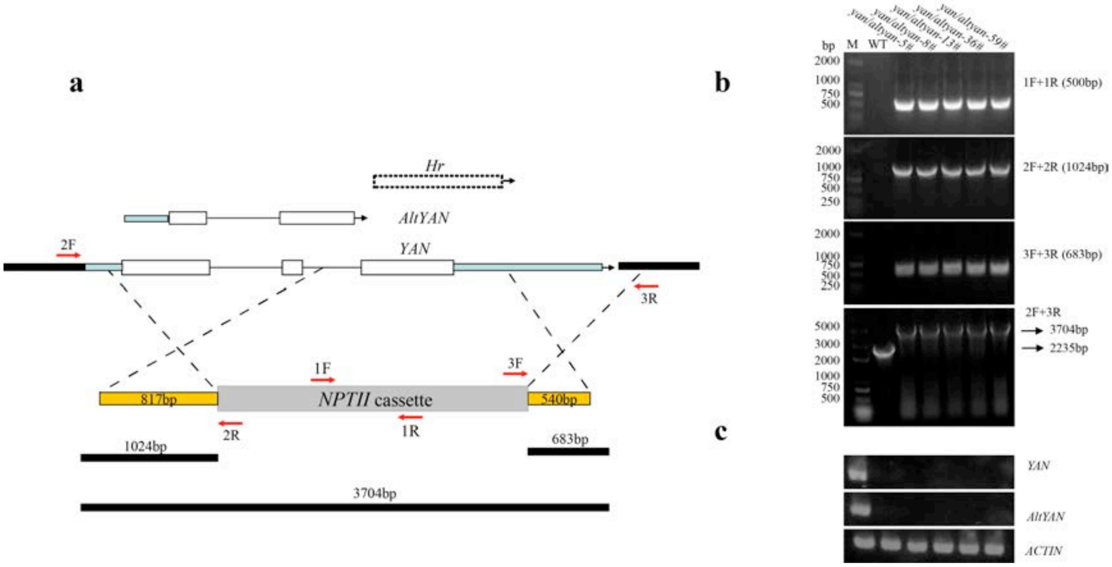

Supplementary Figure 10. Generation of *yan/altyan* knockout mutants and molecular identification. (**a**) Schematic diagram of *YAN/AltYAN* gene disruption construct. The positions of the primers used for PCR analyses of wild-type plants and mutants are indicated with arrows. (**b**) The transformants were tested by PCR on genomic DNA using primer pairs shown in schematic diagram. (**c**) *YAN* and *AltYAN* transcriptions were detected in wild-type plants and *yan/altyan* mutants using semi-quantitative RT-PCR. Primers specific to *ACTIN* were used as the control gene. As shown in the figure, *YAN* and *AltYAN* transcriptions were abolished in *yan/altyan* knockout mutants.

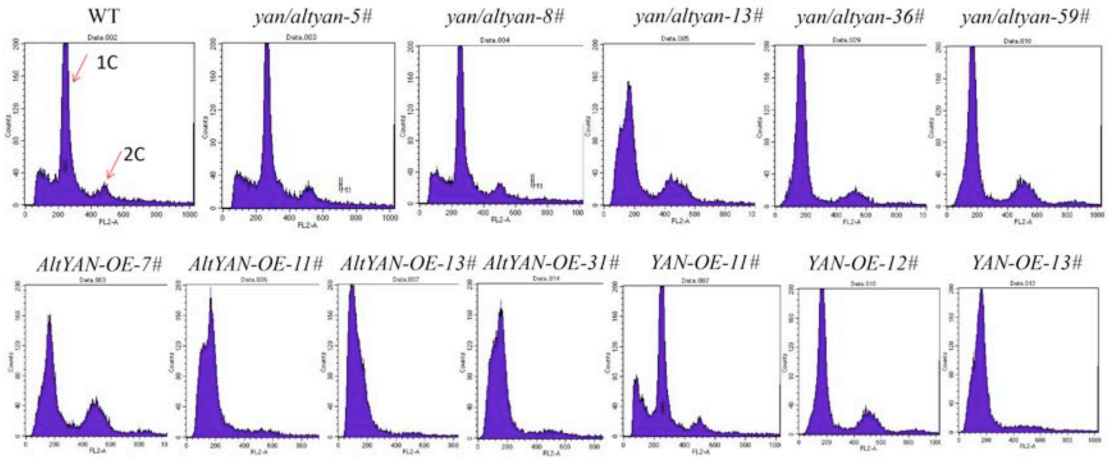

Supplementary Figure 11. Chromosome ploidy analyses. The chromosome ploidy of 2-week old plants was detected using flow cytometry.

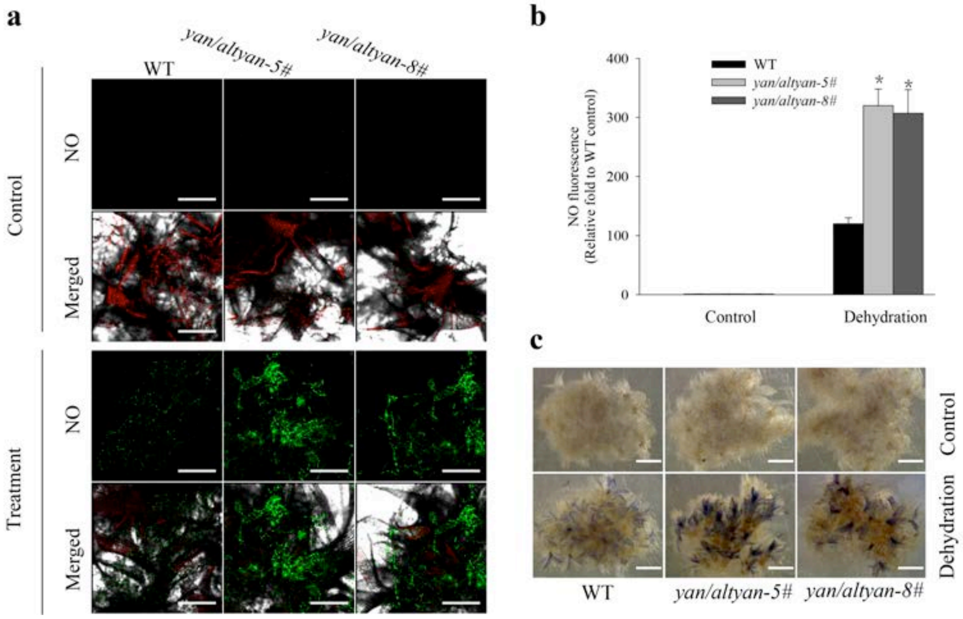

Supplementary Figure 12. Nitric oxide (NO) and superoxide anion accumulation in wild-type plants and *yan/altyan* knockout mutants. Two-week old *P. patens* plants were dehydrated for 20 hours and transferred into sterile water for 1 hour. Plants were then transferred onto standard medium for recovery. Superoxide anions were detected following two days of recovery. (**a**) Toxic NO accumulation in wild-type plants and *yan/altyan.* More toxic NO can be observed in *yan/altyan* mutants than wild-type plants under water stress. NO (green), chlorophyll (red). Scale bar = 1 mm. (**b**) Quantitative analyses of NO fluorescence in *yan/altyan* mutants and wild-type plant using image J software. Values show means and standard deviations of three biological replicates for the wild-type and two independent mutants. Asterisks indicate a statistically significant difference compared with wild-type plants based on a two-tailed student's *t*-test (p<0.05). Scale bar = 2 mm. (**c**) Comparison of superoxide anion accumulation in *yan/altyan* mutants and wild-type plants under dehydration stress. Dark brown areas show superoxide anions.

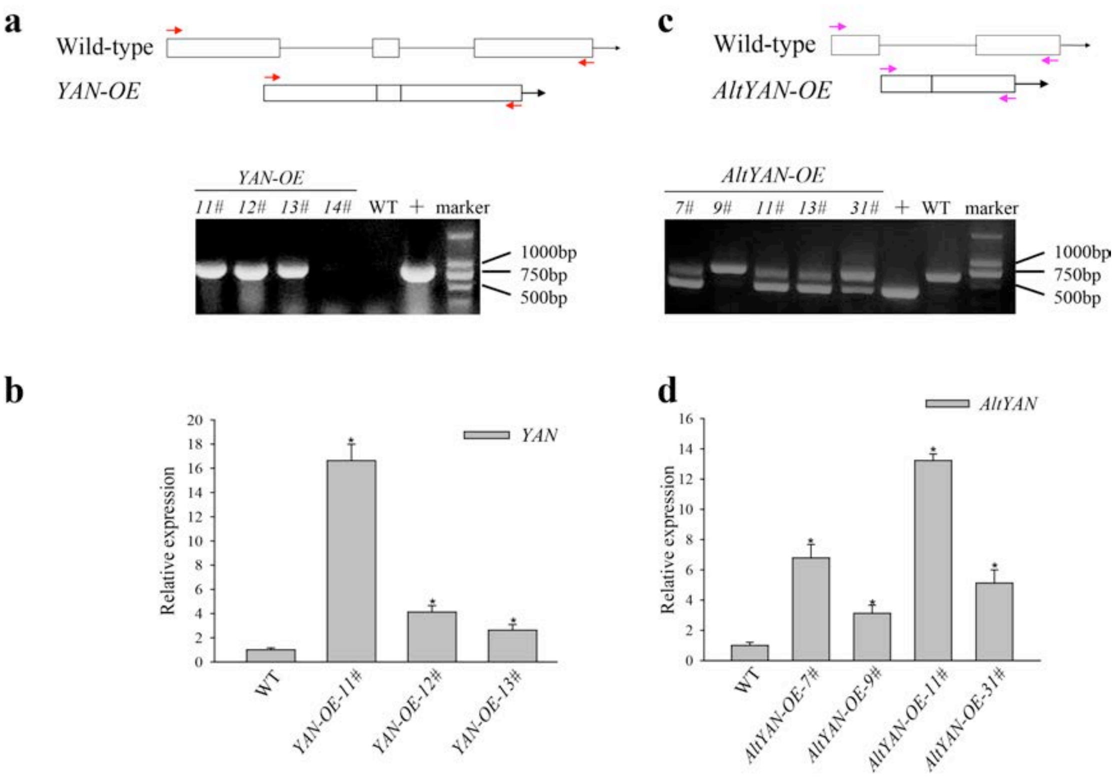

Supplementary Figure 13. Genotyping of *YAN-OE* and *AltYAN-OE* lines. (**a**) Genomic PCR was performed using DNA templates of *YAN-OE,* wild-type (negative control), and *pPOG1-YAN* (positive control). A primer pair designed to amplify full-length CDS of *YAN* was used. (**b**) Quantitative PCR was used to detect the expression level of *YAN* in *YAN-OE* lines. Values show means and standard deviations of three biological replicates for the wild-type and three *YAN-OE* lines. Asterisks indicate a statistically significant difference compared with wild-type plants based on a two-tailed student's *t*-test (p<0.05). (**c**) Genomic PCR was performed using DNA templates of *AltYAN-OE,* wild-type (negative control), and *pPOG1-AltYAN* (positive control). A primer pair designed to amplify full-length CDS of *AltYAN* was used. (**d**) Quantitative PCR was used to detect the expression level of *AltYAN* in *AltYAN-OE* lines. Values show means and standard deviations of three biological replicates for the wild-type and four *AltYAN-OE* lines. Asterisks indicate a statistically significant difference compared with wild-type plants based on a two-tailed student's *t*-test (p<0.05).

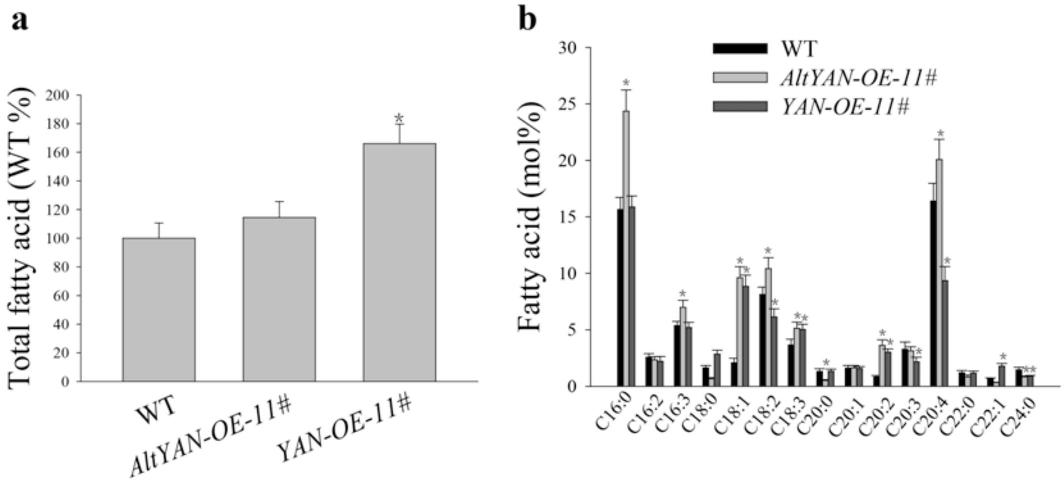

Supplementary Figure 14. Quantification of fatty acids in wild-type, *AltYAN-OE* and *YAN-OE* lines. Ten-week old plants were used to detect the composition and total amount of fatty acids. Values show means and standard deviations of three biological replicates for the wild-type, *AltYAN-OE-11#* and *YAN-OE-11#*. Asterisks indicate a statistically significant difference compared with wild-type plants based on a two-tailed student's *t*-test (p<0.05). (**a**) Total fatty acid content in the wild-type, *AltYAN-OE* and *YAN-OE* lines. **(b**) Comparison of fatty acid amount and composition in wild-type, *AltYAN-OE* and *YAN-OE* lines.

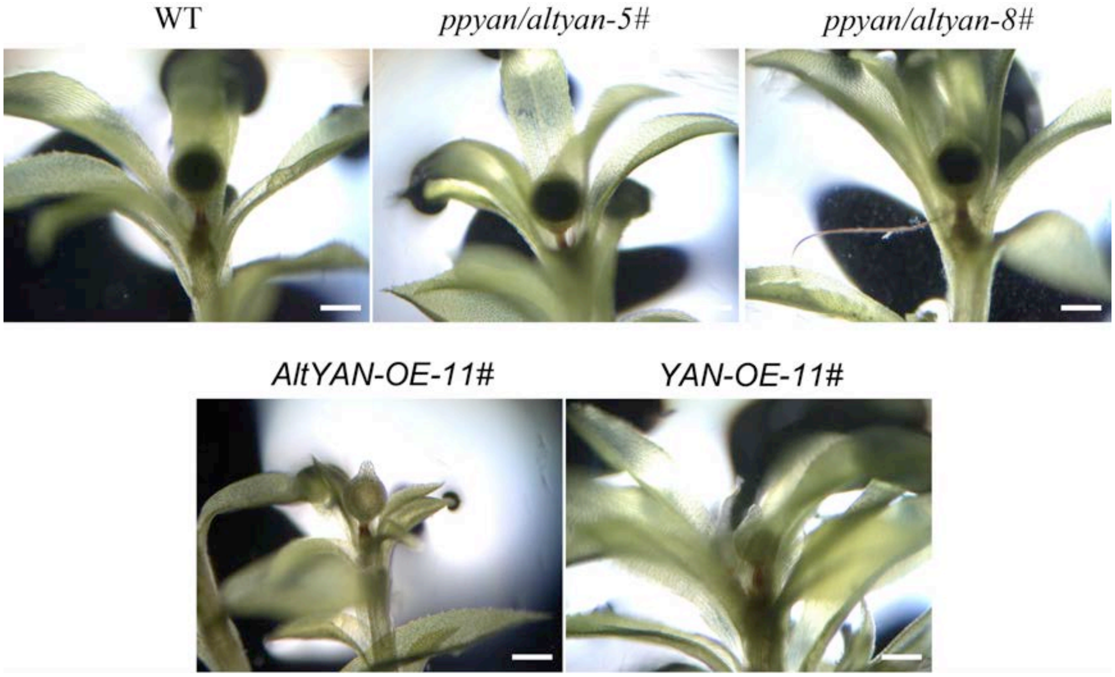

Supplementary Figure 15. Sporophytes of the wild-type and mutants. Plants were grown for 8 weeks at 25 °C before they were transferred to a short-day regime at 15 °C. After 4 weeks of sporophyte induction, visible sporophytes were photographed. Scale bar = 100 µm.

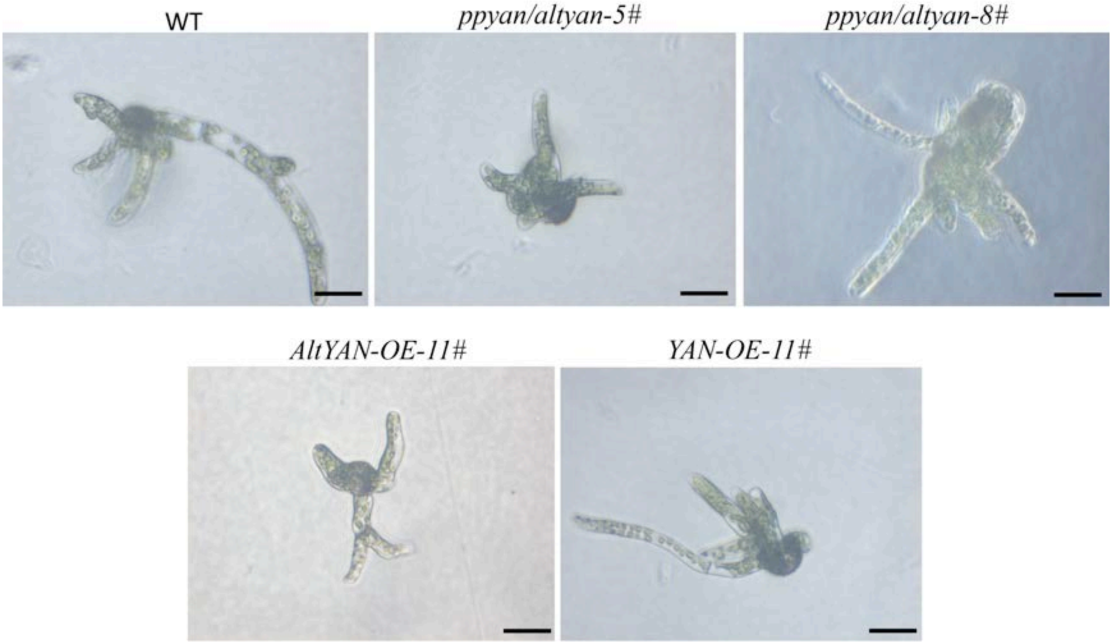

Supplementary Figure 16. Germination of spores from wild-type and mutant plants of *Physcomitrella patens*. Mature sporangia were sterilized using 10% sodium hypocholorite solution for 10 minutes, then washed 5 times using sterilized water. The sporangia were crushed using the tip of a pipet. The released spores were spread on BCDAT medium. The germinated spores were photographed after 4 days of incubation. Scale bar = 50 µm.

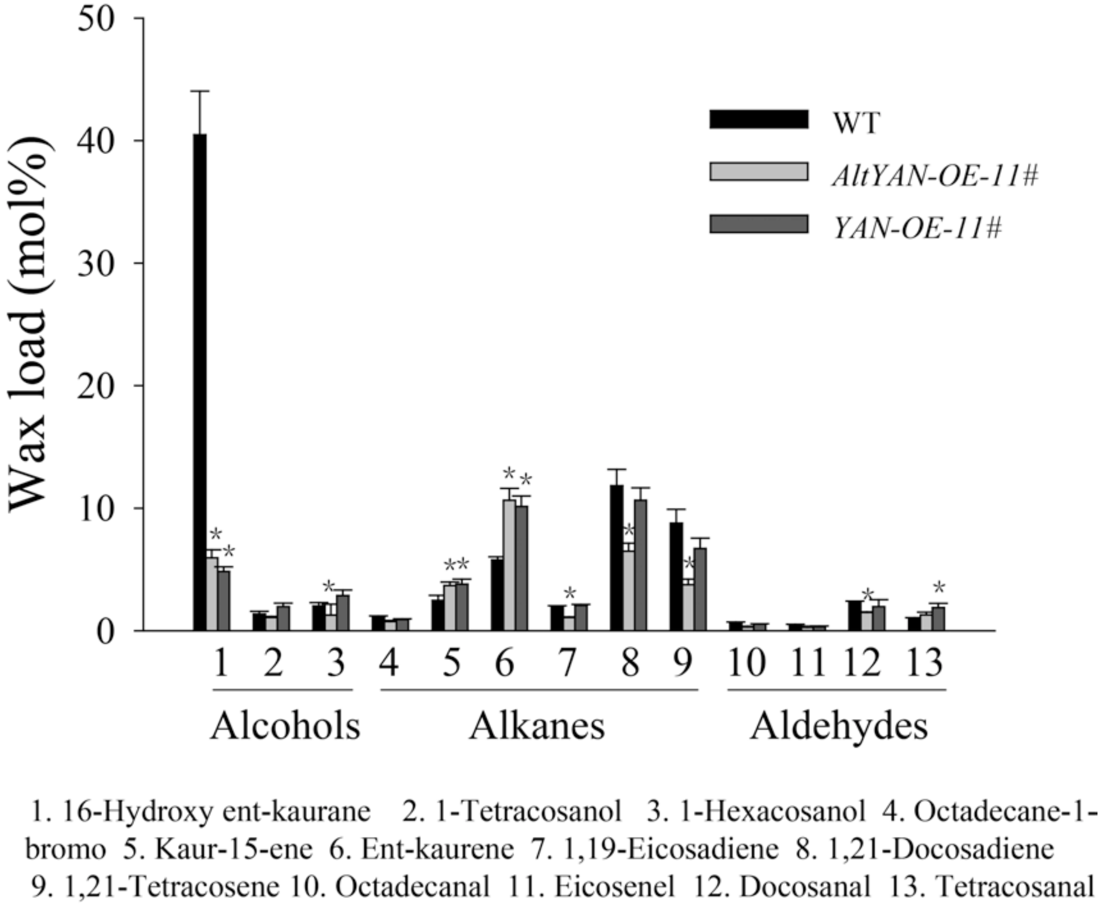

Supplementary Figure 17. Quantification of wax in wild-type, *AltYAN-OE* and *YAN-OE* lines. Ten-week old plants were used to detect the composition of wax. Values show means and standard deviations of three biological replicates for the wild-type, *AltYAN-OE-11#* and *YAN-OE-11#*. Asterisks indicate a statistically significant difference compared with wild-type plants based on a two-tailed student's *t*-test ($p<0.05$).

a

Tor10224 Gametophyte rehydration Library Syntrichia ruralis cDNA, mRNA sequence.
Sequence ID: CN200553.1 **Length:** 501 **Number of Matches:** 1

Range 1: 1 to 444 GenBank Graphics ▼ Next Match ▲ Previous Match

Score	Expect	Method	Identities	Positives	Gaps	Frame
192 bits(489)	7e-60	Compositional matrix adjust.	84/148(57%)	110/148(74%)	0/148(0%)	+1

```
Query  47   KWFNQLVWELSRHMVGEELVVYPLLDAMGPEGKEMADKDRADHQKVKKELEEIHRLSDPG  106
            +WFNQ VWE+SRH V EELV+YPL++A G  G E+A++ R DHQ+ K  LEE+ R +DP
Sbjct  1    RWFNQFVWEISRHAVSEELVLYPLIEAKGQRGSELANQSRTDHQQTKDILEELQRTTDPD  180

Query  107  LFEATLDRIMQDLRQHTNKEETQDLEYLKQHADKKSLENAAKAFKYGKKMAPTRPHPGIP  166
            LFE  +D++M +LR+H  KEE++DL YLK+H  +   E A  AF  GK MAPTRPH  +P
Sbjct  181  LFEQRMDKMMAELREHIRKEESEDLVYLKEHVSQDGREAAGTAFSLGKTMAPTRPHASVP  360

Query  167  NRSAALEAALAFFVTPLDKLRDMFTPFP  194
            N+S A+EAAL   +TP+DKLRD+FTPFP
Sbjct  361  NKSVAIEAALGLLLTPMDKLRDIFTPFP  444
```

b

EST00039 mRNP Lambda ZapII Express Library Syntrichia ruralis cDNA clone mRNP39 3', mRNA sequence.
Sequence ID: AI305074.1 **Length:** 323 **Number of Matches:** 1

Range 1: 10 to 108 GenBank Graphics ▼ Next Match ▲ Previous Match

Score	Expect	Method	Identities	Positives	Gaps	Frame
42.7 bits(99)	0.014	Compositional matrix adjust.	18/33(55%)	24/33(72%)	0/33(0%)	+1

```
Query  150  FKYGKKMAPTRPHPGIPNRSAALEAALAFFVTP  182
             + G++   PT+PH  +PN+ AALEAAL  FVTP
Sbjct  10   VRVGEEDVPTKPHAAVPNKPAALEAALGLFVTP  108
```

LIBRARY

Lib Name:	LIBEST_001531 mRNP Lambda ZapII Express Library
Organism:	Syntrichia ruralis
Tissue type:	Desiccated Gametophytes
Vector:	pBluescriptII
R. Site 1:	EcoRI
R. Site 2:	XhoI
Description:	mRNA derived from the polysomal mRNP fraction of desiccated Tortula ruralis gametophytes, directionally cloned cDNA (lambda ZAPII) and in vivo excised to pBluescript (Stratagene)

c

SLA006H09_34729 An expressed sequence tag (EST) collection from the resurrection plant Selaginella lepidophylla Selaginella lepidophylla cDNA clone SLA006H09 5, mRNA sequence.
Sequence ID: BM402674.1 **Length:** 590 **Number of Matches:** 1

Range 1: 249 to 590 GenBank Graphics ▼ Next Match ▲ Previous Match

Score	Expect	Method	Identities	Positives	Gaps	Frame
125 bits(313)	7e-33	Compositional matrix adjust.	58/114(51%)	82/114(71%)	0/114(0%)	+3

```
Query  14   DLMDEIKHDHREFEEFYTNYKRAYAAGDEKEAEKWFNQLVWELSRHMVGEELVVYPLLDA  73
            D++++I  DH E +++Y  YK A    D++EA KWFNQ VWE+SRH V EELV+YPLL+
Sbjct  249  DIIEKITTDHGELDDYYKKYKSAVKGRDKEEALKWFNQFVWEVSRHSVSEELVLYPLLER  428

Query  74   MGPEGKEMADKDRADHQKVKKELEEIHRLSDPGLFEATLDRIMQDLRQHTNKEE  127
             G +GKE+ADK R+DH KVK+ L E+  ++DP  FE  +D +M++LR+H   EE
Sbjct  429  FGDKGKELADKSRSDHHKVKEYLAEMQSINDPLEFEPKMDEMMKELREHIKMEE  590
```

An expressed sequence tag (EST) collection from the resurrection plant Selaginella lepidophylla

Identifiers	BioSample: SAMN00170085; EST: LIBEST_010176
Organism	Selaginella lepidophylla<br>cellular organisms; Eukaryota; Viridiplantae; Streptophyta; Streptophytina; Embryophyta; Tracheophyta; Lycopodiopsida; Selaginellales; Selaginellaceae; Selaginella
Attributes	**development stage** adult<br>**tissue** microphyll fronds undergoing desiccation for 2.5 h<br>**re_1** EcoRI<br>**v_type** Phagemid<br>**re_2** XhoI<br>**vector** Lambda Uni-Zap XR, Bluescript SK-
Description	Library construction was performed according to manufacture's (Stratagene, Inc.) recommended protocol for the Lambda UniZapXR vector and cDNA synthesis kit.
Submission	University of Nevada, Cushman JC; 2002-01-22

Accession: SAMN00170085 ID: 170085
EST

Supplementary Figure 18. *Hr* genes are induced by dehydration and rehydration in two resurrection plants, the twisted moss *Syntrichia ruralis* and the spikemoss *Selaginella lepidophylla*. The Hr protein sequence from *Sphagnum fallax* (Phytozome identifier Sphfalx0048s0034.1) was used as query to perform TBLASTN search of NCBI dbEST database. **(a)** Alignment of *Sphagnum* Hr protein sequences with a translated sequence of *S. ruralis* rehydration transcript. **(b)** Alignment of *Sphagnum* Hr protein sequences with the translated sequence of another *S. ruralis* transcript, with the associated biosample record indicating that the sample was under desiccation. **(c)** Alignment of *Sphagnum* Hr protein sequences with a translated sequence of *S. lepidophylla* EST, with the associated biosample record indicating that the sample was under desiccation for 2.5 hours.

Supplementary Table 1. TBLASTN result of 1KP database using a *Sphagnum* Hr protein sequence as query; Query=Sphfalx0048s0034.1 peptide (212 amino acids); E-value cutoff = 0.01.

Sequences producing significant alignments:	Score (Bits)	E Value
scaffold-GOWD-2080086-Sphagnum_lescurii	285	7e-95
scaffold-RDOO-2010340-Racomitrium_varium	227	4e-70
scaffold-WOGB-2015424-Andreaea_rupestris	219	4e-68
scaffold-FFPD-2009361-Ceratodon_purpureus	221	9e-68
scaffold-RDOO-2116375-Racomitrium_varium	218	3e-67
scaffold-VMXJ-2128859-Leucobryum_albidum	220	7e-67
scaffold-KEFD-2005771-Encalypta_streptocarpa	214	3e-66
scaffold-ABCD-2074154-Niphotrichum_elongatum	213	1e-65
scaffold-RDOO-2116980-Racomitrium_varium	211	5e-64
scaffold-HRWG-2013098-Buxbaumia_aphylla	207	1e-63
scaffold-BPSG-2019772-Scouleria_aquatica	205	3e-62
scaffold-WOGB-2092690-Andreaea_rupestris	204	8e-62
scaffold-LGDQ-2041893-Selaginella_apoda	199	2e-61
scaffold-TXVB-2071911-Lunularia_cruciata	199	4e-60
scaffold-GRKU-2004786-Syntrichia_princeps	193	3e-59
scaffold-JKAA-2008718-Selaginella_wallacei	199	3e-59
scaffold-ABCD-2010707-Niphotrichum_elongatum	196	4e-59
scaffold-JKAA-2008719-Selaginella_wallacei	199	6e-59
scaffold-NGTD-2098183-Dicranum_scoparium	196	1e-58
scaffold-JMXW-2003398-Bryum_argenteum	187	1e-56
scaffold-ZYCD-2004286-Selaginella_acanthonota	196	1e-56
scaffold-JMXW-2003397-Bryum_argenteum	187	3e-56
scaffold-JMXW-2003396-Bryum_argenteum	186	6e-56
scaffold-ZZOL-2052205-Selaginella_stauntoniana	185	5e-55
scaffold-ZYCD-2004285-Selaginella_acanthonota	196	8e-55
scaffold-ORKS-2011502-Philonotis_fontana	191	5e-53
scaffold-GRKU-2012644-Syntrichia_princeps	179	7e-53
scaffold-PKOX-2012929-Isoetes_tegetiformans	181	1e-52
scaffold-GRKU-2012643-Syntrichia_princeps	180	1e-52
scaffold-VMXJ-2124089-Leucobryum_albidum	173	3e-51
scaffold-RDOO-2109788-Racomitrium_varium	169	9e-51
scaffold-FITN-2089742-Treubia_lacunose	177	9e-51
scaffold-PYHZ-2006808-Isoetes_sp	177	2e-50
scaffold-PKOX-2096679-Isoetes_tegetiformans	175	4e-50

scaffold-TMAJ-2173065-Neckera_douglasii	161	2e-47
scaffold-YPIC-2134561-Microstegium_vimineum	149	5e-42
scaffold-RXRQ-2128249-Phaeoceros_carolinianus-sporophyte	145	5e-41
scaffold-WCZB-2013633-Phaeoceros_carolinianus-gametophyte	146	5e-41
scaffold-JMXW-2040177-Bryum_argenteum	146	6e-41
scaffold-WNGH-2006956-Aulacomnium_heterostichum	145	3e-40
scaffold-OFTV-2128708-Barbilophozia_barbata	144	1e-39
scaffold-CMEQ-2082481-Orthotrichum_lyellii	144	2e-39
scaffold-JBGU-2011382-Amaranthus_palmeri	143	2e-39
scaffold-YEPO-2071721-cf._Physcomicromitrium_sp	140	6e-39
scaffold-WCZB-2114770-Phaeoceros_carolinianus-gametophyte	142	4e-38
scaffold-RXRQ-2022078-Phaeoceros_carolinianus-sporophyte	144	4e-38
scaffold-IRBN-2152420-Scapania_nemorosa	139	1e-37
scaffold-RGKI-2054671-Leucobryum_glaucum	134	4e-37
scaffold-JKAA-2174841-Selaginella_wallacei	137	4e-37
scaffold-RXRQ-2132940-Phaeoceros_carolinianus-sporophyte	136	3e-36
scaffold-FFPD-2008086-Ceratodon_purpureus	131	4e-36
scaffold-FAJB-2053787-Paraphymatoceros_hallii	137	8e-36
scaffold-RXRQ-2018681-Phaeoceros_carolinianus-sporophyte	136	2e-35
scaffold-GRKU-2004785-Syntrichia_princeps	132	4e-35
scaffold-YBQN-2122714-Odontoschisma_prostratum	130	9e-35
scaffold-JHFI-2105756-Pellia_neesiana	129	2e-34
scaffold-CMEQ-2081739-Orthotrichum_lyellii	129	5e-34
scaffold-TGKW-2009714-Frullania	126	3e-33
scaffold-LNSF-2063071-Hypnum_subimponens	125	4e-33
scaffold-KJZG-2059461-Asplenium_platyneuron	129	5e-33
scaffold-LNSF-2008214-Hypnum_subimponens	127	7e-33
scaffold-IRBN-2150099-Scapania_nemorosa	125	8e-33
scaffold-NOKI-2092089-Lindsaea_linearis	128	1e-32
scaffold-ABCD-2011017-Niphotrichum_elongatum	129	1e-32
scaffold-NWQC-2149828-Plagiochila_asplenioides	126	2e-32
scaffold-IRBN-2150967-Scapania_nemorosa	125	2e-32
scaffold-JKAA-2177860-Selaginella_wallacei	127	3e-32
scaffold-GSXD-2062693-Myriopteris_eatonii	124	5e-32
scaffold-OFTV-2123041-Barbilophozia_barbata	121	9e-32
scaffold-MZLD-2123763-Ligustrum_sinense	124	1e-31
scaffold-DCDT-2072974-Cheilanthes_arizonica	125	1e-31
scaffold-KJZG-2058634-Asplenium_platyneuron	124	2e-31
scaffold-HNDZ-2129005-Cystopteris_utahensis	122	3e-31
scaffold-JBGU-2121651-Amaranthus_palmeri	123	3e-31
scaffold-LNSF-2008215-Hypnum_subimponens	122	4e-31

scaffold-JKAA-2170971-Selaginella_wallacei	119	8e-31
scaffold-OCZL-2010080-Homalosorus_pycnocarpos	122	1e-30
scaffold-ABCD-2009871-Niphotrichum_elongatum	122	1e-30
scaffold-IGUH-2161180-Schwetschkeopsis_fabronia	120	1e-30
scaffold-WNGH-2085370-Aulacomnium_heterostichum	121	3e-30
scaffold-NWQC-2005566-Plagiochila_asplenioides	120	4e-30
scaffold-NWQC-2005567-Plagiochila_asplenioides	118	9e-30
scaffold-CQPW-2020236-Anemia_tomentosa	119	3e-29
scaffold-CPKP-2097340-Lophophora_williamsii	114	1e-28
scaffold-WFBF-2000168-Podophyllum_peltatum	114	2e-28
scaffold-JKAA-2167480-Selaginella_wallacei	111	5e-28
scaffold-NRWZ-2091968-Metzgeria_crassipilis	110	1e-27
scaffold-XDDT-2012577-Argyrochosma_nivea	112	2e-27
scaffold-IRBN-2142735-Scapania_nemorosa	110	2e-27
scaffold-RDOO-2109148-Racomitrium_varium	106	4e-26
scaffold-WOGB-2081836-Andreaea_rupestris	103	1e-25
scaffold-OSHQ-2044949-Sarcandra_glabra	106	2e-25
scaffold-ZTHV-2000232-Atrichum_angustatum	103	2e-24
scaffold-NRWZ-2009763-Metzgeria_crassipilis	99.4	2e-23
scaffold-WTDE-2023700-Johnsonia_pubescens	99.8	2e-23
scaffold-IGUH-2154130-Schwetschkeopsis_fabronia	99.4	2e-23
scaffold-SALZ-2065380-Pittosporum_resiniferum-fruit	100	8e-23
scaffold-WCZB-2102726-Phaeoceros_carolinianus-gametophyte	96.3	2e-22
scaffold-CHJJ-2127518-Lejeuneaceae_sp	96.7	2e-22
scaffold-CIEA-2049265-Juncus_inflexus	98.6	2e-22
scaffold-NGTD-2090279-Dicranum_scoparium	96.3	3e-22
scaffold-IRBN-2139892-Scapania_nemorosa	95.9	5e-22
scaffold-VQYB-2010950-Neurachne_lanigera	95.5	2e-21
scaffold-ABCD-2064447-Niphotrichum_elongatum	93.6	2e-21
scaffold-OCWZ-2051463-Dioscorea_villosa	97.1	5e-21
scaffold-PSHB-2008862-Lantana_camara	94.0	7e-21
scaffold-KEFD-2050257-Encalypta_streptocarpa	91.7	9e-21
scaffold-KEFD-2054814-Encalypta_streptocarpa	92.8	1e-20
scaffold-VMXJ-2107202-Leucobryum_albidum	89.0	5e-20
scaffold-YWNF-2008347-Hedwigia_ciliata	90.9	1e-19
scaffold-RGKI-2054107-Leucobryum_glaucum	88.2	2e-19
scaffold-TMAJ-2001356-Neckera_douglasii	92.0	2e-19
scaffold-YWNF-2008346-Hedwigia_ciliata	91.7	4e-19
scaffold-RTMU-2149348-Calypogeia_fissa	86.7	7e-19
scaffold-HATH-2079510-Aristida_stricta	87.0	9e-19
scaffold-ZXJO-2012394-Hemionitis_arifolia	87.0	3e-18

scaffold-YPIC-2132251-Microstegium_vimineum	86.3	4e-18
scaffold-WVMY-2126909-Phlox_drummondii	86.3	4e-18
scaffold-ABIJ-2031254-Selaginella_lepidophylla	83.2	9e-18
scaffold-MVSE-2025297-Griselinia_littoralis	85.5	9e-18
scaffold-RDOO-2015219-Racomitrium_varium	85.5	1e-17
scaffold-IRBN-2122784-Scapania_nemorosa	80.9	7e-17
scaffold-VMXJ-2115109-Leucobryum_albidum	81.6	7e-17
scaffold-UEEN-2146356-Forestiera_segregata	80.5	1e-16
scaffold-YPIC-2127709-Microstegium_vimineum	81.3	1e-16
scaffold-NWQC-2113770-Plagiochila_asplenioides	80.1	1e-16
scaffold-IRBN-2155004-Scapania_nemorosa	85.1	2e-16
scaffold-WNGH-2012483-Aulacomnium_heterostichum	83.2	2e-16
scaffold-YPIC-2115414-Microstegium_vimineum	79.0	3e-16
scaffold-POPJ-2014900-Pteris_vittata	80.5	4e-16
scaffold-PKOX-2008033-Isoetes_tegetiformans	59.7	9e-16
scaffold-IGUH-2105402-Schwetschkeopsis_fabronia	77.0	1e-15
scaffold-QMWB-2052150-Anomodon_attenuatus	78.6	1e-15
scaffold-NGTD-2089101-Dicranum_scoparium	79.0	1e-15
scaffold-BGXB-2069679-Plagiomnium_insigne	78.6	1e-15
scaffold-OCWZ-2048666-Dioscorea_villosa	79.3	2e-15
scaffold-IRBN-2002456-Scapania_nemorosa	78.6	2e-15
scaffold-TVSH-2057737-Bituminaria_bituminosa	77.0	3e-15
scaffold-XWHK-2033525-Funaria	75.9	3e-15
scaffold-NGTD-2072576-Dicranum_scoparium	75.9	3e-15
scaffold-NDUV-2084566-Vittaria_appalachiana	75.9	6e-15
scaffold-TMAJ-2176774-Neckera_douglasii	78.6	1e-14
scaffold-WTDE-2120279-Johnsonia_pubescens	75.5	1e-14
scaffold-WPUV-2050890-Oenothera_grandis-MTJ_135	74.7	3e-14
scaffold-ZENX-2075599-Neurachne_alopecuroidea	73.6	4e-14
scaffold-VMXJ-2101389-Leucobryum_albidum	72.8	6e-14
scaffold-XWDM-2024866-XWDM-Cystopteris_fragilis-2_samples_combined	62.0	9e-14
scaffold-CPKP-2083294-Lophophora_williamsii	72.0	2e-13
scaffold-CVEG-2117220-Azolla_cf._caroliniana	72.4	2e-13
scaffold-RTMU-2146607-Calypogeia_fissa	72.0	3e-13
scaffold-PYHZ-2058919-Isoetes_sp	65.9	3e-13
scaffold-TMAJ-2146025-Neckera_douglasii	70.1	8e-13
scaffold-UPMJ-2080149-Pseudolycopodiella_caroliniana	73.6	1e-12
scaffold-ZTHV-2078155-Atrichum_angustatum	59.3	1e-12
scaffold-RTMU-2082020-Calypogeia_fissa	68.6	1e-12
scaffold-CMEQ-2067654-Orthotrichum_lyellii	68.9	2e-12
scaffold-RTMU-2159248-Calypogeia_fissa	70.9	2e-12

scaffold-KEFD-2052864-Encalypta_streptocarpa	70.5	2e-12
scaffold-CMEQ-2063716-Orthotrichum_lyellii	67.4	4e-12
scaffold-YPIC-2024478-Microstegium_vimineum	70.1	5e-12
scaffold-HATH-2073870-Aristida_stricta	68.2	6e-12
scaffold-PMRP-2036296-Cannabis_sativa-stem	70.1	1e-11
scaffold-BJSW-2066960-BJSW-Cannabis_sativa-4_samples_combined	70.1	1e-11
scaffold-IMZV-2080047-IMZV-Oenothera_grandis-5_samples_combined	67.0	2e-11
scaffold-RXRQ-2131384-Phaeoceros_carolinianus-sporophyte	69.7	2e-11
scaffold-OCWZ-2044044-Dioscorea_villosa	66.2	3e-11
scaffold-CHJJ-2130531-Lejeuneaceae_sp	68.2	3e-11
scaffold-GKAG-2079692-Huperzia_lucidula	65.1	5e-11
scaffold-IGUH-2160559-Schwetschkeopsis_fabronia	68.6	6e-11
scaffold-CHJJ-2012733-Lejeuneaceae_sp	68.6	6e-11
scaffold-KRUQ-2055797-Porella_navicularis	63.2	1e-10
scaffold-VMXJ-2081240-Leucobryum_albidum	63.5	1e-10
scaffold-JKAA-2154702-Selaginella_wallacei	64.3	1e-10
scaffold-JSAG-2054274-Masdevallia_yuangensis	62.8	2e-10
scaffold-FITN-2084753-Treubia_lacunose	65.9	2e-10
scaffold-GRKU-2069920-Syntrichia_princeps	63.5	3e-10
scaffold-ABCD-2067800-Niphotrichum_elongatum	64.3	5e-10
scaffold-IGUH-2157080-Schwetschkeopsis_fabronia	64.7	6e-10
scaffold-RTMU-2014569-Calypogeia_fissa	46.6	6e-10
scaffold-NRWZ-2079339-Metzgeria_crassipilis	62.4	8e-10
scaffold-NRWZ-2092900-Metzgeria_crassipilis	64.3	1e-09
scaffold-LHLE-2056411-Cystopteris_fragilis	61.6	1e-09
scaffold-HATH-2079179-Aristida_stricta	63.2	1e-09
scaffold-UUHD-2077091-Porella_pinnata	60.5	1e-09
scaffold-LNSF-2055857-Hypnum_subimponens	62.0	2e-09
scaffold-FFPD-2029090-Ceratodon_purpureus	59.7	2e-09
scaffold-TMAJ-2012211-Neckera_douglasii	62.8	2e-09
scaffold-MIRS-2073569-Climacium_dendroides	61.2	3e-09
scaffold-HPXA-2104167-Ptilidium_pulcherrimum	60.5	3e-09
scaffold-CHJJ-2000052-Lejeuneaceae_sp	63.2	4e-09
scaffold-GRKU-2071783-Syntrichia_princeps	60.8	5e-09
scaffold-VMXJ-2123744-Leucobryum_albidum	63.2	5e-09
scaffold-HVBQ-2112238-Tetraphis_pellucida	60.5	6e-09
scaffold-QMWB-2055650-Anomodon_attenuatus	62.0	6e-09
scaffold-HPXA-2127478-Ptilidium_pulcherrimum	62.0	7e-09
scaffold-WOGB-2084446-Andreaea_rupestris	60.5	7e-09
scaffold-JKAA-2078739-Selaginella_wallacei	57.8	1e-08
scaffold-AEXY-2130519-Blasia_sp	60.8	1e-08

scaffold-JKAA-2165817-Selaginella_wallacei	60.5	1e-08
scaffold-PSHB-2043956-Lantana_camara	58.2	1e-08
scaffold-NGTD-2089411-Dicranum_scoparium	60.1	1e-08
scaffold-JMXW-2034222-Bryum_argenteum	58.5	2e-08
scaffold-TMAJ-2003433-Neckera_douglasii	58.9	2e-08
scaffold-NRWZ-2085378-Metzgeria_crassipilis	58.9	2e-08
scaffold-KEFD-2044179-Encalypta_streptocarpa	57.4	4e-08
scaffold-GRRW-2048048-Grevillea_robusta	57.8	4e-08
scaffold-ABCD-2058843-Niphotrichum_elongatum	57.4	5e-08
scaffold-PSHB-2039572-Lantana_camara	55.5	9e-08
scaffold-XNXF-2001698-Dendrolycopodium_obscurum	53.5	1e-07
scaffold-IRBN-2103858-Scapania_nemorosa	55.5	2e-07
scaffold-TMWO-2076697-Papaver_bracteatum-bulb	54.7	2e-07
scaffold-SSDU-2123337-SSDU-Papaver_bracteatum-3_samples_combined	54.7	2e-07
scaffold-VMXJ-2120453-Leucobryum_albidum	57.4	2e-07
scaffold-JHCN-2084608-Oxalis_sp	57.0	3e-07
scaffold-NRWZ-2088588-Metzgeria_crassipilis	56.2	3e-07
scaffold-UAXP-2066247-Gyrostemon_ramulosus	55.5	3e-07
scaffold-IGUH-2005101-Schwetschkeopsis_fabronia	57.4	3e-07
scaffold-NRWZ-2089194-Metzgeria_crassipilis	56.2	4e-07
scaffold-RTMU-2146256-Calypogeia_fissa	55.5	4e-07
scaffold-VMXJ-2077554-Leucobryum_albidum	53.9	5e-07
scaffold-OFTV-2003852-Barbilophozia_barbata	57.4	5e-07
scaffold-ZXJO-2005224-Hemionitis_arifolia	55.8	6e-07
scaffold-ABIJ-2034612-Selaginella_lepidophylla	55.5	8e-07
scaffold-YEPO-2030533-cf._Physcomicromitrium_sp	52.8	8e-07
scaffold-WOGB-2008726-Andreaea_rupestris	55.8	9e-07
scaffold-CHJJ-2116743-Lejeuneaceae_sp	53.9	1e-06
scaffold-IRBN-2005941-Scapania_nemorosa	56.2	2e-06
scaffold-IRBN-2005942-Scapania_nemorosa	56.2	2e-06
scaffold-IRBN-2076572-Scapania_nemorosa	52.0	2e-06
scaffold-ABCD-2014511-Niphotrichum_elongatum	51.2	3e-06
scaffold-OFTV-2087677-Barbilophozia_barbata	42.0	3e-06
scaffold-HPXA-2008638-Ptilidium_pulcherrimum	38.9	3e-06
scaffold-RDOO-2089861-Racomitrium_varium	52.0	4e-06
scaffold-IRBN-2155117-Scapania_nemorosa	55.1	5e-06
scaffold-IGUH-2005100-Schwetschkeopsis_fabronia	53.9	5e-06
scaffold-YPIC-2124849-Microstegium_vimineum	52.4	6e-06
scaffold-HPXA-2105951-Ptilidium_pulcherrimum	51.6	7e-06
scaffold-MROH-2036845-Thelypteris_acuminata	52.0	7e-06
scaffold-ZSAB-2180975-Hoheria_angustifolia	52.0	9e-06

scaffold-YCKE-2055091-Notholaena_montieliae	52.8	9e-06
scaffold-TMAJ-2120109-Neckera_douglasii	50.8	9e-06
scaffold-LRTN-2087772-Monotropa_uniflora	51.6	1e-05
scaffold-GRKU-2044556-Syntrichia_princeps	49.7	1e-05
scaffold-CHJJ-2119525-Lejeuneaceae_sp	51.2	2e-05
scaffold-NGTD-2066643-Dicranum_scoparium	49.7	2e-05
scaffold-CMEQ-2082860-Orthotrichum_lyellii	53.1	3e-05
scaffold-WOGB-2051835-Andreaea_rupestris	48.5	3e-05
scaffold-MVSE-2190933-Griselinia_littoralis	49.3	4e-05
scaffold-PSHB-2043518-Lantana_camara	43.1	5e-05
scaffold-HATH-2002294-Aristida_stricta	49.7	5e-05
scaffold-JKAA-2053839-Selaginella_wallacei	48.1	5e-05
scaffold-YWNF-2020517-Hedwigia_ciliata	47.8	5e-05
scaffold-YWNF-2020516-Hedwigia_ciliata	47.8	5e-05
scaffold-FROP-2070730-FROP-Epifagus_virginiana-2_samples_combined	49.3	6e-05
scaffold-WCZB-2085267-Phaeoceros_carolinianus-gametophyte	48.5	6e-05
scaffold-BJSW-2012126-BJSW-Cannabis_sativa-4_samples_combined	50.1	7e-05
scaffold-WFBF-2003972-Podophyllum_peltatum	51.2	7e-05
scaffold-WOGB-2008722-Andreaea_rupestris	49.3	8e-05
scaffold-VMXJ-2036090-Leucobryum_albidum	47.0	9e-05
scaffold-OQWW-2161174-Davallia_fejeensis	47.4	1e-04
scaffold-YPIC-2134240-Microstegium_vimineum	50.1	2e-04
scaffold-KRUQ-2090857-Porella_navicularis	47.8	2e-04
scaffold-NGTD-2062919-Dicranum_scoparium	47.0	2e-04
scaffold-OXYP-2078939-Sideroxylon_reclinatum	46.6	2e-04
scaffold-KEFD-2052641-Encalypta_streptocarpa	48.5	2e-04
scaffold-XRCX-2095148-Aster_tataricus	48.5	2e-04
scaffold-CHJJ-2127168-Lejeuneaceae_sp	48.5	3e-04
scaffold-JKAA-2061455-Selaginella_wallacei	45.8	3e-04
scaffold-CVEG-2094112-Azolla_cf._caroliniana	46.2	4e-04
scaffold-OQWW-2161093-Davallia_fejeensis	45.8	4e-04
scaffold-JKAA-2008453-Selaginella_wallacei	47.8	4e-04
scaffold-LNSF-2045802-Hypnum_subimponens	46.2	5e-04
scaffold-NRWZ-2086705-Metzgeria_crassipilis	47.4	5e-04
scaffold-FAJB-2050601-Paraphymatoceros_hallii	47.8	6e-04
scaffold-NDUV-2080635-Vittaria_appalachiana	45.8	7e-04
scaffold-VMXJ-2028561-Leucobryum_albidum	44.7	7e-04
scaffold-TGKW-2021476-Frullania	44.3	0.001
scaffold-EWXK-2077938-Thyrsopteris_elegans	44.7	0.001
scaffold-RDOO-2025299-Racomitrium_varium	43.9	0.001
scaffold-KEFD-2043942-Encalypta_streptocarpa	45.1	0.001

scaffold-VMXJ-2028149-Leucobryum_albidum	43.5	0.002
scaffold-VBMM-2023838-Anomodon_rostratus	43.5	0.002
scaffold-JKAA-2129507-Selaginella_wallacei	43.9	0.002
scaffold-PMRP-2006102-Cannabis_sativa-stem	45.4	0.003
scaffold-NRWZ-2069162-Metzgeria_crassipilis	43.9	0.003
scaffold-NDUV-2096264-Vittaria_appalachiana	45.8	0.004
scaffold-WOGB-2064377-Andreaea_rupestris	43.1	0.005
scaffold-NRWZ-2047801-Metzgeria_crassipilis	42.0	0.009
scaffold-CHJJ-2131097-Lejeuneaceae_sp	44.7	0.009

Supplementary Table 2. Taxonomic report of TBLASTN search of *nr* database using a translated charophyte sequence (ISGT-2038756) as query. The sequence was retrieved from 1KP and translated into six frames. The match to Hr protein sequences was used as query to search the nr databases. E-value cutoff = 1.

Organism	Blast Name	Score	Number of Hits	Description
cellular organisms			167	
.Bacteria	bacteria		159	
..Proteobacteria	proteobacteria		143	
...Alphaproteobacteria	a-proteobacteria		99	
....Rhodospirillales	a-proteobacteria		11	
.....Rhodospirillaceae	a-proteobacteria		4	
......Rhodospirillaceae bacterium CCH5-H10	a-proteobacteria	50.4	1	Rhodospirillaceae bacterium CCH5-H10 hits
......Phaeospirillum fulvum	a-proteobacteria	46.6	1	Phaeospirillum fulvum hits
......Phaeospirillum fulvum MGU-K5	a-proteobacteria	39.7	1	Phaeospirillum fulvum MGU-K5 hits
......Dongia sp. URHE0060	a-proteobacteria	37.4	1	Dongia sp. URHE0060 hits
.....Rhodospirillales bacterium URHD0017	a-proteobacteria	47.8	4	Rhodospirillales bacterium URHD0017 hits
.....Reyranella massiliensis	a-proteobacteria	40.4	1	Reyranella massiliensis hits
.....Rhodospirillales bacterium SCN 65-16	a-proteobacteria	40.0	1	Rhodospirillales bacterium SCN 65-16 hits
.....Enhydrobacter aerosaccus	a-proteobacteria	37.0	1	Enhydrobacter aerosaccus hits
....Sphingobium cloacae	a-proteobacteria	47.0	2	Sphingobium cloacae hits
....Sphingobium baderi	a-proteobacteria	46.6	2	Sphingobium baderi hits
....Sphingobium chungbukense	a-proteobacteria	46.2	2	Sphingobium chungbukense hits
....Sphingobium japonicum	a-proteobacteria	46.2	2	Sphingobium japonicum hits
....Sphingobium japonicum UT26S	a-proteobacteria	46.2	1	Sphingobium japonicum UT26S hits
....Sphingobium	a-proteobacteria	46.2	2	Sphingobium hits
....Sphingobium chinhatense IP26	a-proteobacteria	46.2	1	Sphingobium chinhatense IP26 hits
....Sphingobium sp. HDIP04	a-proteobacteria	46.2	1	Sphingobium sp. HDIP04 hits
....Sphingobium lucknowense F2	a-proteobacteria	46.2	1	Sphingobium lucknowense F2 hits
....Sphingomonadaceae	a-proteobacteria	46.2	2	Sphingomonadaceae hits
....Sphingomonas sp. BHC-A	a-proteobacteria	46.2	1	Sphingomonas sp. BHC-A hits
....Sphingobium indicum B90A	a-proteobacteria	46.2	1	Sphingobium indicum B90A hits
....Sphingobium yanoikuyae	a-proteobacteria	44.7	4	Sphingobium yanoikuyae hits
....Sphingobium sp. YBL2	a-proteobacteria	44.7	1	Sphingobium sp. YBL2 hits
....Sphingobium sp. AM	a-proteobacteria	44.7	1	Sphingobium sp. AM hits
....Sphingobium sp. 22B	a-proteobacteria	44.7	1	Sphingobium sp. 22B hits
....Sphingomonas paucimobilis	a-proteobacteria	44.7	3	Sphingomonas paucimobilis hits
....Sphingomonas sp. MEA3-1	a-proteobacteria	44.7	1	Sphingomonas sp. MEA3-1 hits
....alpha proteobacterium LLX12A	a-proteobacteria	43.1	1	alpha proteobacterium LLX12A hits
....Novosphingobium sp. Rr 2-17	a-proteobacteria	43.1	2	Novosphingobium sp. Rr 2-17 hits
....Caulobacter sp. K31	a-proteobacteria	43.1	2	Caulobacter sp. K31 hits
....Sphingobium sp. DC-2	a-proteobacteria	42.4	1	Sphingobium sp. DC-2 hits
....Caulobacter sp. Root343	a-proteobacteria	42.4	2	Caulobacter sp. Root343 hits
....Caulobacter henricii	a-proteobacteria	42.4	1	Caulobacter henricii hits
....Brevundimonas viscosa	a-proteobacteria	42.4	2	Brevundimonas viscosa hits
....Sphingomonas koreensis	a-proteobacteria	42.0	3	Sphingomonas koreensis hits
....Sphingobium quisquiliarum	a-proteobacteria	42.0	1	Sphingobium quisquiliarum hits
....Sphingobium quisquiliarum P25	a-proteobacteria	42.0	1	Sphingobium quisquiliarum P25 hits
....Sphingobium sp. MI1205	a-proteobacteria	41.2	2	Sphingobium sp. MI1205 hits
....Sphingomonas sp. Root241	a-proteobacteria	41.2	2	Sphingomonas sp. Root241 hits
....Novosphingobium panipatense	a-proteobacteria	41.2	1	Novosphingobium panipatense hits
....Sphingobium herbicidovorans	a-proteobacteria	41.2	1	Sphingobium herbicidovorans hits
....Sphingobium herbicidovorans NBRC 16415	a-proteobacteria	41.2	1	Sphingobium herbicidovorans NBRC 16415 hits
....Sphingomonas pruni	a-proteobacteria	40.8	1	Sphingomonas pruni hits
....Sphingobium sp. YL23	a-proteobacteria	40.0	1	Sphingobium sp. YL23 hits
....Sphingomonas sp. Ndbn-20	a-proteobacteria	40.0	1	Sphingomonas sp. Ndbn-20 hits
....Sphingomonas sp. URHD0057	a-proteobacteria	40.0	1	Sphingomonas sp. URHD0057 hits
....Sphingobium sp. SYK-6	a-proteobacteria	40.0	2	Sphingobium sp. SYK-6 hits
....Sphingomonas	a-proteobacteria	39.7	1	Sphingomonas hits
....Sphingomonas sp. Root1294	a-proteobacteria	39.7	1	Sphingomonas sp. Root1294 hits
....Sphingomonas sp. Root50	a-proteobacteria	39.7	1	Sphingomonas sp. Root50 hits
....Sphingomonas sp. Root720	a-proteobacteria	39.7	1	Sphingomonas sp. Root720 hits
....Sphingomonas sp. MCT13	a-proteobacteria	39.7	2	Sphingomonas sp. MCT13 hits

....Sphingomonas sp. JJ-A5	a-proteobacteria	39.3	2	Sphingomonas sp. JJ-A5 hits
....Sphingobium sp. EP60837	a-proteobacteria	39.3	2	Sphingobium sp. EP60837 hits
....Methylocystis sp. SC2	a-proteobacteria	39.3	1	Methylocystis sp. SC2 hits
....Sphingobium sp. Ant17	a-proteobacteria	38.5	2	Sphingobium sp. Ant17 hits
....Caulobacterales bacterium 32-69-10	a-proteobacteria	38.5	1	Caulobacterales bacterium 32-69-10 hits
....Sphingomonas sp. 28-66-16	a-proteobacteria	38.5	1	Sphingomonas sp. 28-66-16 hits
....Sphingomonas sp. UNC305MFCol5.2	a-proteobacteria	38.1	1	Sphingomonas sp. UNC305MFCol5.2 hits
....Novosphingobium sp. MD-1	a-proteobacteria	38.1	1	Novosphingobium sp. MD-1 hits
....Sphingobium sp. YR657	a-proteobacteria	38.1	2	Sphingobium sp. YR657 hits
....Sphingobium yanoikuyae ATCC 51230	a-proteobacteria	38.1	1	Sphingobium yanoikuyae ATCC 51230 hits
....Sphingobium japonicum BiD32	a-proteobacteria	37.4	1	Sphingobium japonicum BiD32 hits
....Brevundimonas	a-proteobacteria	37.0	1	Brevundimonas hits
....Brevundimonas sp. Root1423	a-proteobacteria	37.0	1	Brevundimonas sp. Root1423 hits
....Brevundimonas sp. Root608	a-proteobacteria	37.0	1	Brevundimonas sp. Root608 hits
....Sphingomonas hengshuiensis	a-proteobacteria	37.0	2	Sphingomonas hengshuiensis hits
....Sphingomonas asaccharolytica	a-proteobacteria	37.0	1	Sphingomonas asaccharolytica hits
....Sphingomonas mali	a-proteobacteria	37.0	1	Sphingomonas mali hits
....Sphingobium sp. AP49	a-proteobacteria	36.2	2	Sphingobium sp. AP49 hits
...Burkholderia sp. A27	b-proteobacteria	49.7	2	Burkholderia sp. A27 hits
...Paraburkholderia	b-proteobacteria	47.4	1	Paraburkholderia hits
...Paraburkholderia sediminicola	b-proteobacteria	47.4	1	Paraburkholderia sediminicola hits
...Paraburkholderia terricola	b-proteobacteria	47.4	1	Paraburkholderia terricola hits
...Paraburkholderia terrae	b-proteobacteria	46.2	8	Paraburkholderia terrae hits
...Paraburkholderia terrae BS001	b-proteobacteria	46.6	2	Paraburkholderia terrae BS001 hits
...Burkholderia sp. yr281	b-proteobacteria	46.6	2	Burkholderia sp. yr281 hits
...Paraburkholderia monticola	b-proteobacteria	43.1	2	Paraburkholderia monticola hits
...Mitsuaria sp. 7	b-proteobacteria	42.0	2	Mitsuaria sp. 7 hits
...Burkholderia sp. YR277	b-proteobacteria	41.6	2	Burkholderia sp. YR277 hits
...Aeromonas salmonicida	g-proteobacteria	40.8	4	Aeromonas salmonicida hits
...Nitrosomonas sp. Nm166	b-proteobacteria	40.8	2	Nitrosomonas sp. Nm166 hits
...Burkholderia sp. UYPR1.413	b-proteobacteria	40.8	1	Burkholderia sp. UYPR1.413 hits
...Rhizobacter sp. Root404	b-proteobacteria	40.4	2	Rhizobacter sp. Root404 hits
...Roseateles depolymerans	b-proteobacteria	39.7	2	Roseateles depolymerans hits
...Ramlibacter sp. Leaf400	b-proteobacteria	39.3	2	Ramlibacter sp. Leaf400 hits
...Rhizobacter sp. OV335	b-proteobacteria	38.5	2	Rhizobacter sp. OV335 hits
...Mitsuaria sp. HZ7	b-proteobacteria	37.7	2	Mitsuaria sp. HZ7 hits
...Cellvibrio sp. BR	g-proteobacteria	37.0	2	Cellvibrio sp. BR hits
...Roseateles terrae	b-proteobacteria	36.6	2	Roseateles terrae hits
..Streptomyces sp. F-3	high GC Gram+	40.4	2	Streptomyces sp. F-3 hits
..Actinomadura rubrobrunea	high GC Gram+	40.0	1	Actinomadura rubrobrunea hits
..Phormidium ambiguum	cyanobacteria	39.7	1	Phormidium ambiguum hits
..Phormidium ambiguum IAM M-71	cyanobacteria	39.7	1	Phormidium ambiguum IAM M-71 hits
..Streptomyces sp. TP-A0356	high GC Gram+	39.7	1	Streptomyces sp. TP-A0356 hits
..Streptomyces sp. e14	high GC Gram+	39.3	1	Streptomyces sp. e14 hits
..Oscillatoria sp. PCC 10802	cyanobacteria	38.1	1	Oscillatoria sp. PCC 10802 hits
..Chloroflexi bacterium 54-19	GNS bacteria	38.5	1	Chloroflexi bacterium 54-19 hits
..Nocardia africana	high GC Gram+	37.7	1	Nocardia africana hits
..Cyanothece sp. PCC 7822	cyanobacteria	37.4	2	Cyanothece sp. PCC 7822 hits
..Micromonospora echinaurantiaca	high GC Gram+	36.2	2	Micromonospora echinaurantiaca hits
..Streptomyces alni	high GC Gram+	36.2	2	Streptomyces alni hits
.Gonium pectorale	green algae	47.4	1	Gonium pectorale hits
.Monoraphidium neglectum	green algae	46.2	2	Monoraphidium neglectum hits
.Phlebia centrifuga	basidiomycetes	41.2	1	Phlebia centrifuga hits
.Sphaerobolus stellatus SS14	basidiomycetes	40.4	1	Sphaerobolus stellatus SS14 hits
.Rhizophagus irregularis DAOM 181602	glomeromycetes	38.5	1	Rhizophagus irregularis DAOM 181602 hits
.Rhizophagus irregularis DAOM 197198w	glomeromycetes	38.5	1	Rhizophagus irregularis DAOM 197198w hits
.Gonapodya prolifera JEL478	monoblepharidomycetes	37.7	1	Gonapodya prolifera JEL478 hits

Supplementary Note 1. With Hr protein sequences from various nonvascular plants and green algae as query, the only two hits from seed plants in the NCBI nucleotide sequence database belong to *Quercus suber* (Fagaceae). These two hits were located on two separate scaffolds of the unpublished draft genome sequence of this species, and they share 88% protein sequence identity. Both sequences are significantly more similar to fungal homologs than to other green plant sequences (up to 68% protein sequence identities with fungi versus 36-37% with the seedless vascular plant *Selaginella moellendorffii*). It is unclear whether these two sequences are derived from sequencing contamination or horizontal gene transfer.

The many hemerythrin hits from flowering plants raises a significant concern about contamination in 1KP data. Other than chlorophycean sequences, which can be verified through the scaffold information of their complete genomes, other algal sequences are only sporadically found in various lineages, including red algae (*Glaucosphera vacuolata*, 1KP IDs: RSOF_scaffold_2008604, RSOF_scaffold_2041944, RSOF_scaffold_2004035, RSOF_scaffold_2032925), glaucophytes (*Glaucocystis nostochinearum*, 1KP IDs: POOW_scaffold_2004542, POOW_scaffold_2053347) and charophytes (*Hormidiella sp.*, 1KP IDs: ISGT-scaffold_2038756, ISGT-scaffold_2001291; *Coleochaete irregulares*, 1KP IDs: QPDY-scoffold_2002876, QPDY-scaffold_2002877), and other chlorophytes (*Chlorella* IKP ID: MWAN_scaffold_2004366; *Parachlorella kessleri,* IKP ID: AKCR_scaffold_2002030; *Ignatius tetrasporus,* 1KP ID: KADG_scaffold_2037975; *Botryococcus_sudeticus,* IKP ID: VJDZ_scaffold_2007822). Because the 1KP database only contains plant sequence data, we further searched the *nr* database using the above sequences as query to investigate whether they were indeed similar to chlorophycean and land plant hemerythrins. These analyses showed that the red algal sequences were significantly more similar to fungal homologs than to chlorophycean and land plant sequences. Not hits to red algae were identified, even though multiple red algal genomes (e.g., *Cyanodioschyzon merolae, Chondrus crispus, Galdieria sulphuraria, Porphyridium purpureum*, *Porphyra umbilicalis,* and *Pyropia yezoensis*) have been sequenced and deposited in NCBI. Similarly, the glaucophytes were significantly more similar to fungal and

bacterial sequences than to chlorophycean and land plant sequences (in both sequence coverage and identity).

Of the four charophyte sequences, three (1KP IDs: ISGT-scaffold_2038756, QPDY-scaffold_2002876 and QPDY-scaffold_2002877) shared higher or similar percent identities with bacterial and fungal homologs (see **Supplementary Table 2** above), and one (1KP ID: ISGT-scaffold_2001291) was slightly more similar to chlorophycean sequences. Although many other green algae outside Chlorophyceae (e.g., the charophyte *Klebsormidium nitens,* chlorophytes *Ostreococcus tauri, Ostreococcus lucimarinus, Micromonas pusilla, Cymbomonas tetramitiformis, Coccomyxa subellipsoidea, Chlorella variabilis, Chlorella vulgaris, Auxenochlorella protothecoides, Picochlorum sp., Helicosporidium sp.*) have been sequenced and their data have been deposited in NCBI, no hits from these taxa were detected. Except for *Parachlorella kessleri*, all other chlorophyte sequences also shared a higher sequence coverage and identity with bacterial or fungal homologs, and again no hits from green algae outside Chlorophyceae were detected. The *Parachlorella kessleri* sequence was more similar to a protein sequence of *Chlorella variabilis* that was translated from mRNA data (NCBI accession number: XP_005847154), which in turn was more similar to fungal and bacterial sequences.

Supplementary Note 2. We searched the *Nitella hyalina* transcriptomic data at NCBI (https://trace.ncbi.nlm.nih.gov/Traces/sra/?run=SRR064326), but only two matches of 19-32 nucleotides were found; these two matches were mapped onto different regions (separated by about 470 nucleotides) of the *Hr* CDS (see figure below, top panel). Search of the transcriptomic data of the charophyte *Closterium peracerosum-strigosum-littorale complex* at NCBI (https://www.ncbi.nlm.nih.gov/bioproject?term=PRJNA296352) provided a similar result (also see below, lower panel).

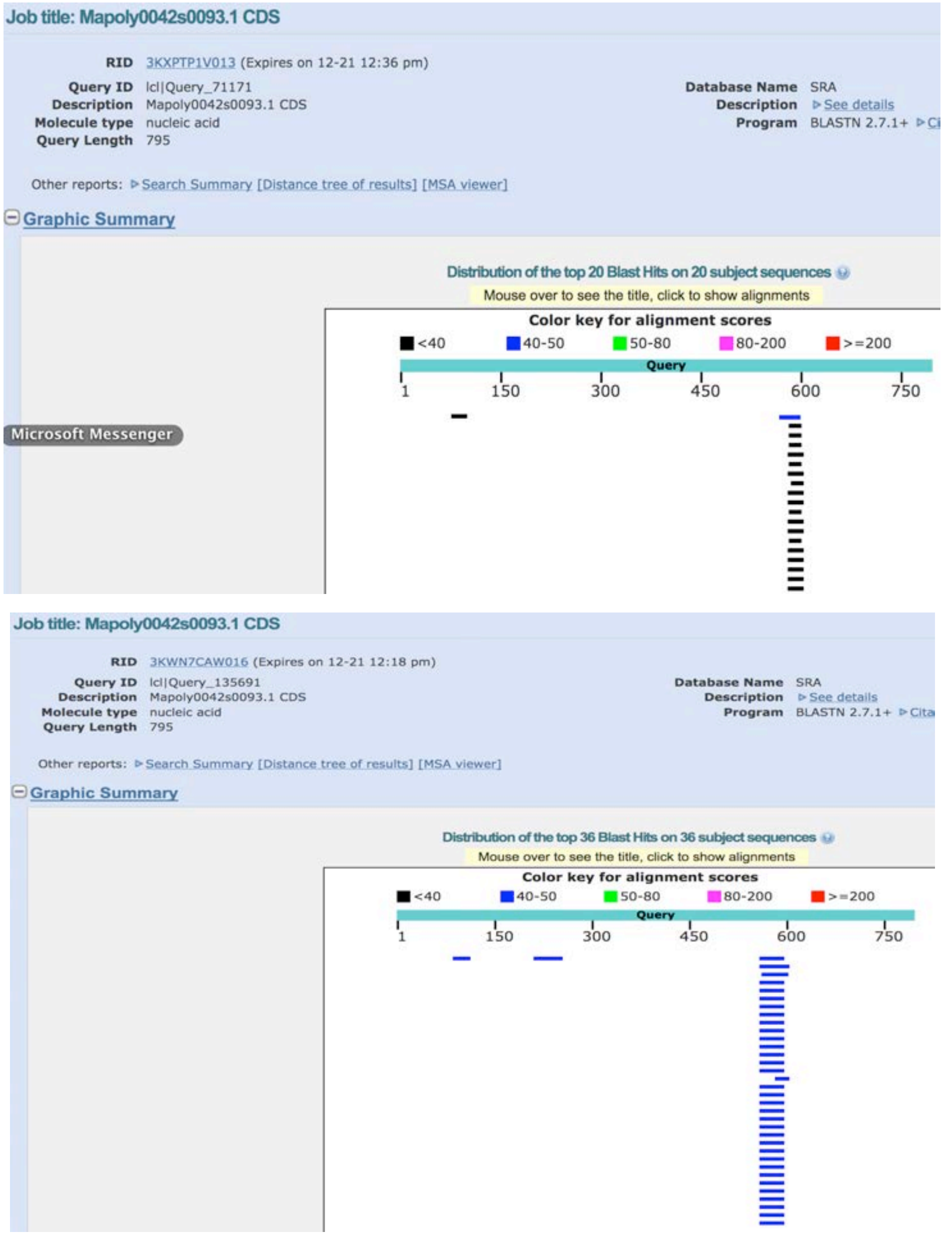

BLASTN search of transcriptomic data of charophytes *Nitella hyalina* (top panel) and *Closterium peracerosum-strigosum-littorale* complex (lower panel) at NCBI. Query is the CDS of *Marchantia polymorpha Hr* (JGI Id: Mapoly0042s0093). Results indicate that only short fragments are mapped to the query.

Because the assessment of sequence homology is more difficult at the level of nucleotide sequences, lack of sufficient coverage to the query in the above search does not necessarily suggest the absence of *Hr* in the two charophytes. Therefore, we further investigated whether the 2-3 charophyte hits were specific to *Hr* (i.e., the HHE domain). To this end, we performed the BLASTN search of NCBI non-redundant nucleotide sequence database (nt) using the same *Marchantia polymorpha Hr* (JGI Id: Mapoly0042s0093) as query. Indeed, the results included hits corresponding to those from the two charophytes (*Nitella hyalina* and *Closterium peracerosum-strigosum-littorale* complex) (see figure below, top panel). However, further inspections of these hits indicated that they were not particular to the *Hr* gene. For instance, the hits to the 670-600 bp region of the query were annotated as part of the genes encoding small integral membrane protein 10-like protein 2A, peroxidase 7-like protein, and myomegalin-like protein; they were found in both flowering plants (*Lupinus angustifolius*, *Vitis venifera*, and *Cucurbita maxima*) and animals (*Crocodylus porosus*, Oncorhynchus mykiss, and *Columba livia*). Similarly, hits to the 150-200 bp region of the query were annotated as genes encoding erythrocyte membrane protein, RP1 like 1 (rp1l1) protein, and retrotransposon Gag like 5 (Rtl5) protein; they could be found in animals (e.g., *Oryzias latipes*, *Heterocephalus glaber*, *Labrus bergylta*) and apicomplexan parasites (*Plasmodium falciparum*). On the other hand, the most significant hits, which also had the longest query coverages, were from *Selaginella moellendorffii*, the fungus *Fusarium verticillioides* and *Physcomitrella patens* (see figure below, lower panel). The former two were part of the *Hr* gene, whereas the later (i.e. *P. patens*) evolved directly from *Hr*. These data suggest that the hits from the two charophytes (*Nitella hyalina* and *Closterium peracerosum-strigosum-littorale* complex) might be associated with genes other than *Hr*.

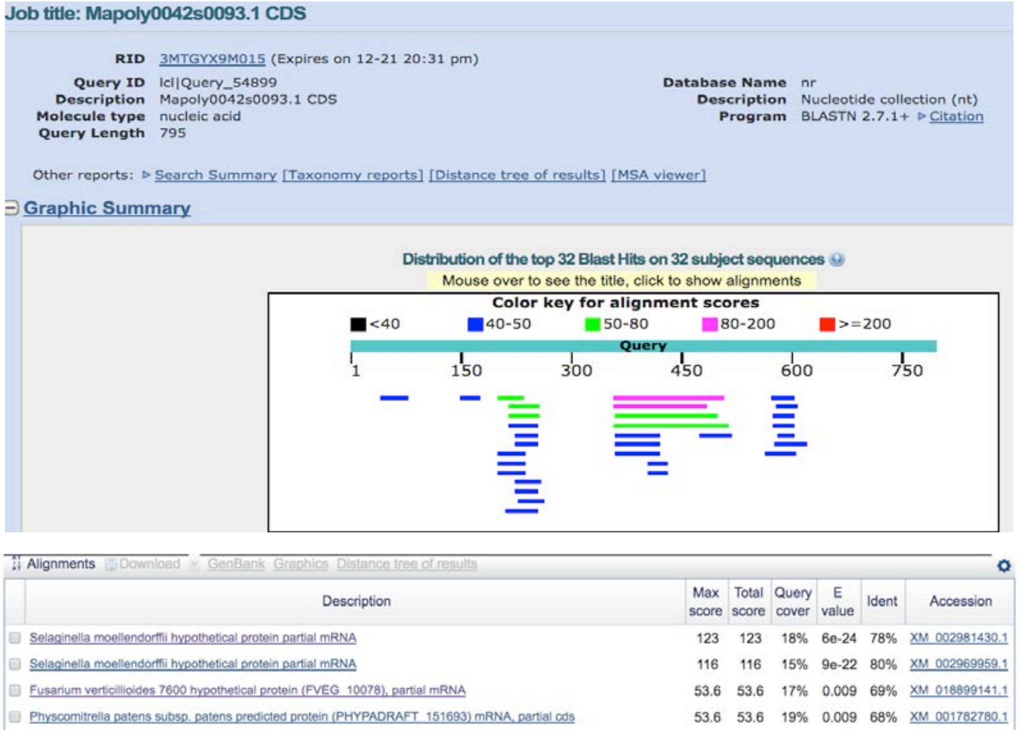
Job title: Mapoly0042s0093.1 CDS

RID 3MTGYX9M015 (Expires on 12-21 20:31 pm)

Query ID lcl|Query_54899
Description Mapoly0042s0093.1 CDS
Molecule type nucleic acid
Query Length 795

Database Name nr
Description Nucleotide collection (nt)
Program BLASTN 2.7.1+ ▷ Citation

Other reports: ▷ Search Summary [Taxonomy reports] [Distance tree of results] [MSA viewer]

Graphic Summary

Alignments Download GenBank Graphics Distance tree of results

Description	Max score	Total score	Query cover	E value	Ident	Accession
Selaginella moellendorffii hypothetical protein partial mRNA	123	123	18%	6e-24	78%	XM_002981430.1
Selaginella moellendorffii hypothetical protein partial mRNA	116	116	15%	9e-22	80%	XM_002969959.1
Fusarium verticillioides 7600 hypothetical protein (FVEG_10078), partial mRNA	53.6	53.6	17%	0.009	69%	XM_018899141.1
Physcomitrella patens subsp. patens predicted protein (PHYPADRAFT_151693) mRNA, partial cds	53.6	53.6	19%	0.009	68%	XM_001782780.1

The following includes uncropped gel images for corresponding Supplementary Figures above.

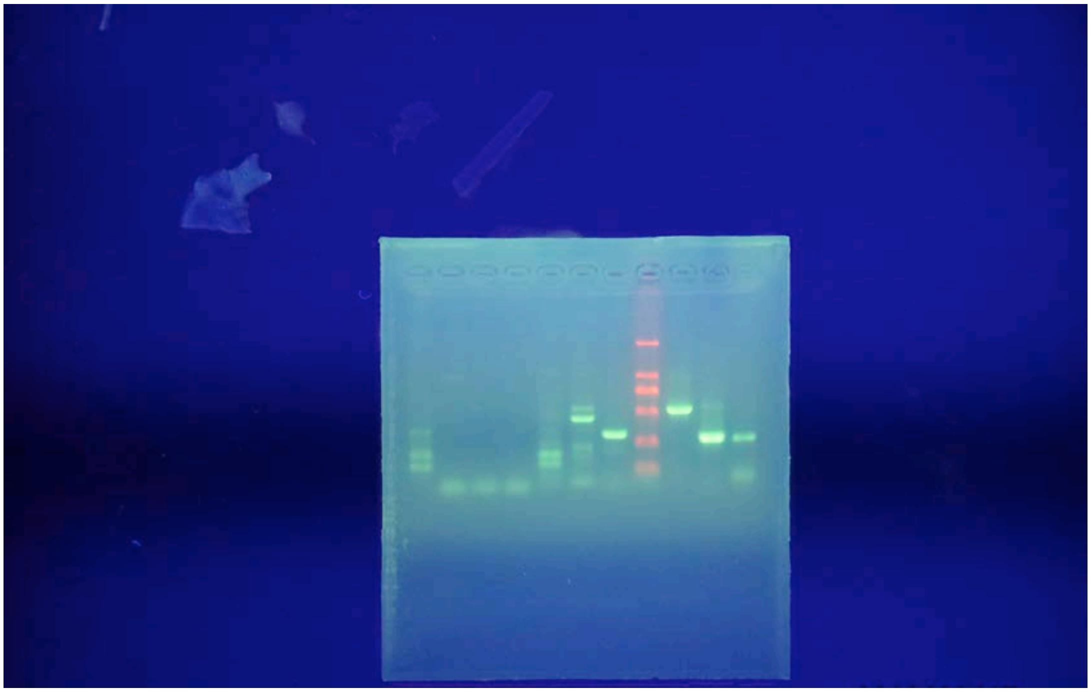

Uncropped gel of supplementary figure 6b

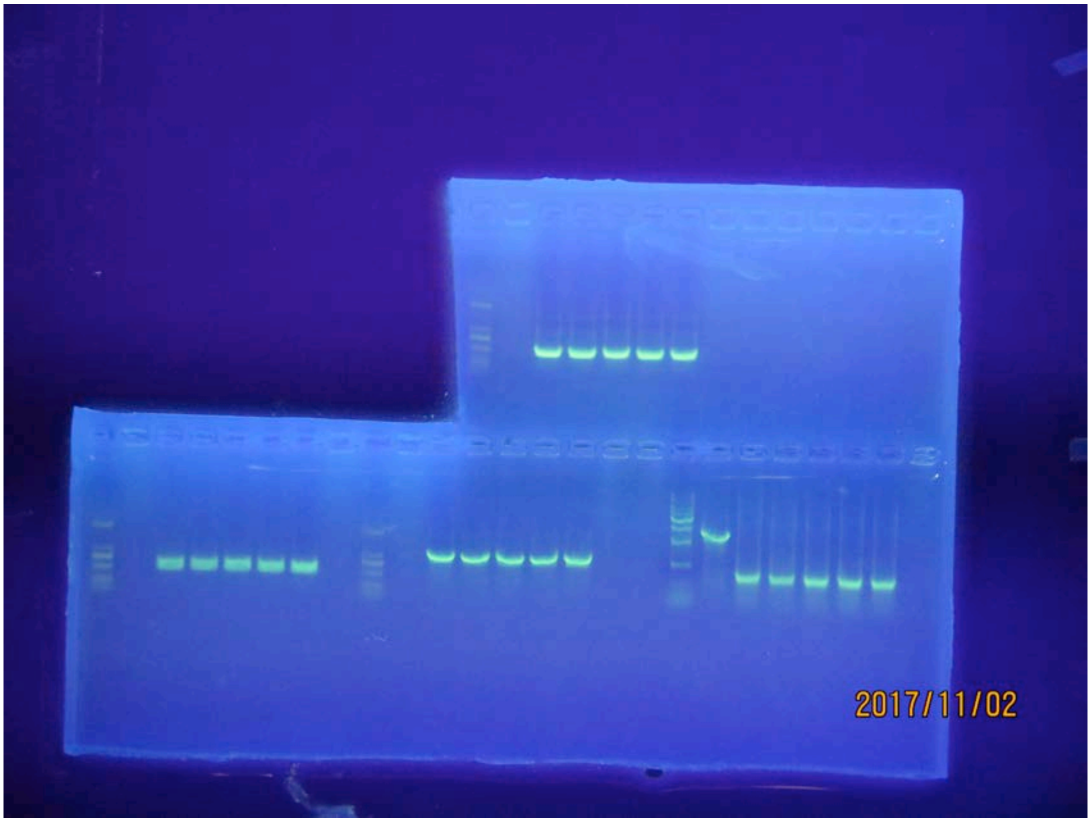

Uncropped gel of supplementary figure 10b

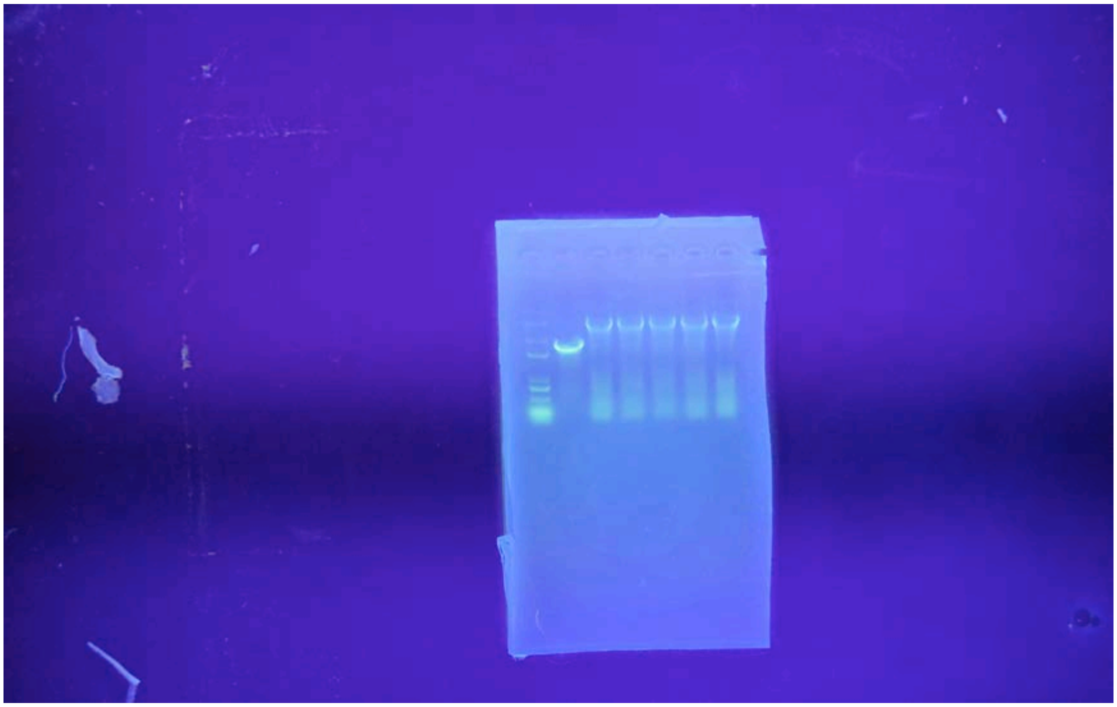

Uncropped gel of supplementary figure 10b

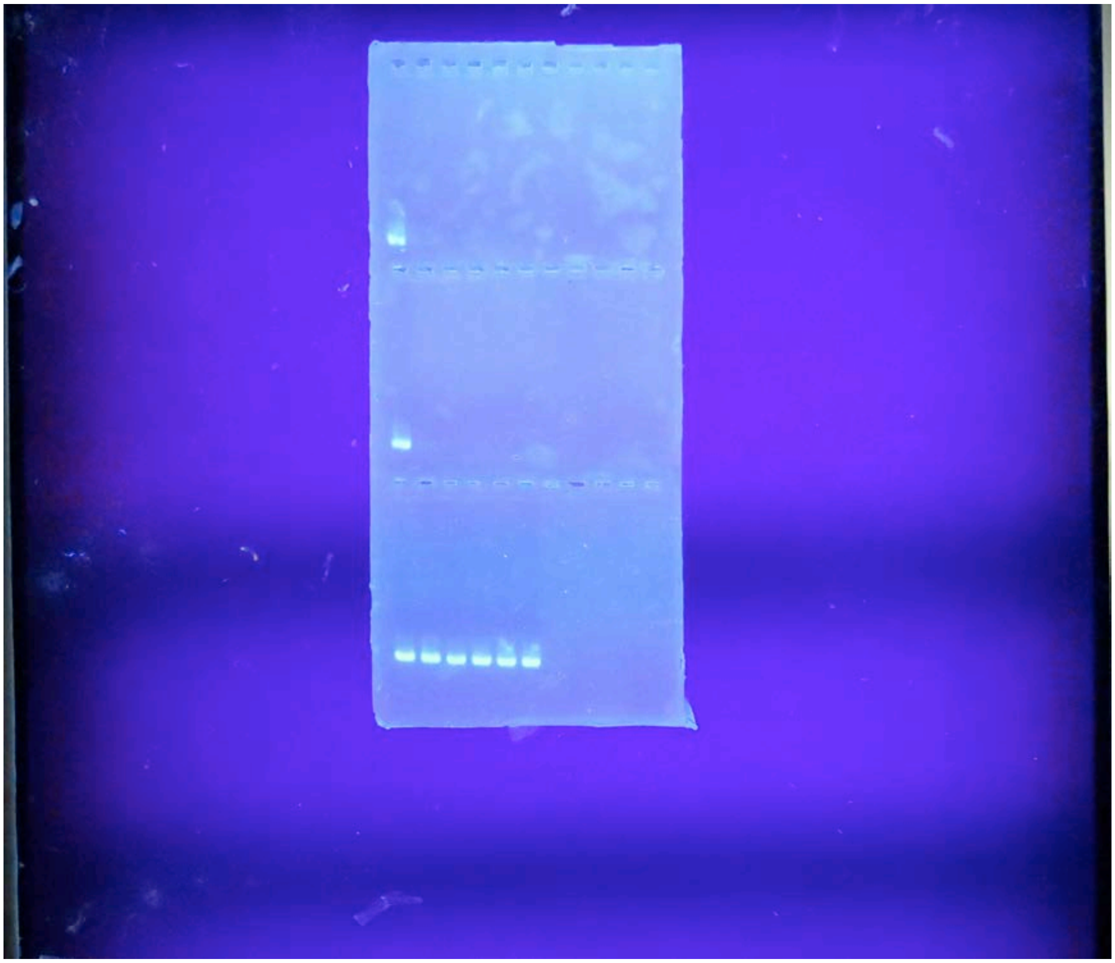

Uncropped gel of supplementary figure 10c

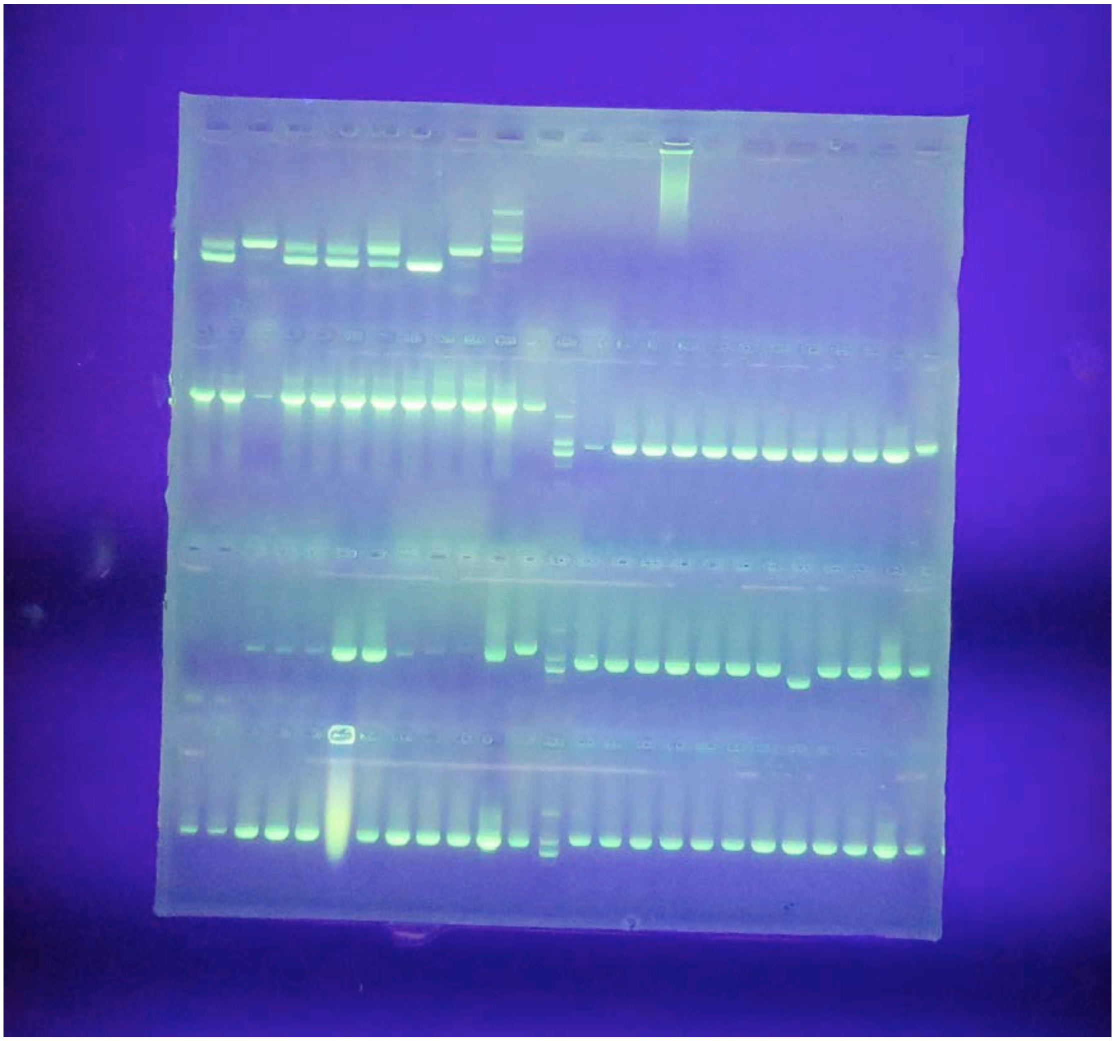

Uncropped gel of supplementary figure 13a

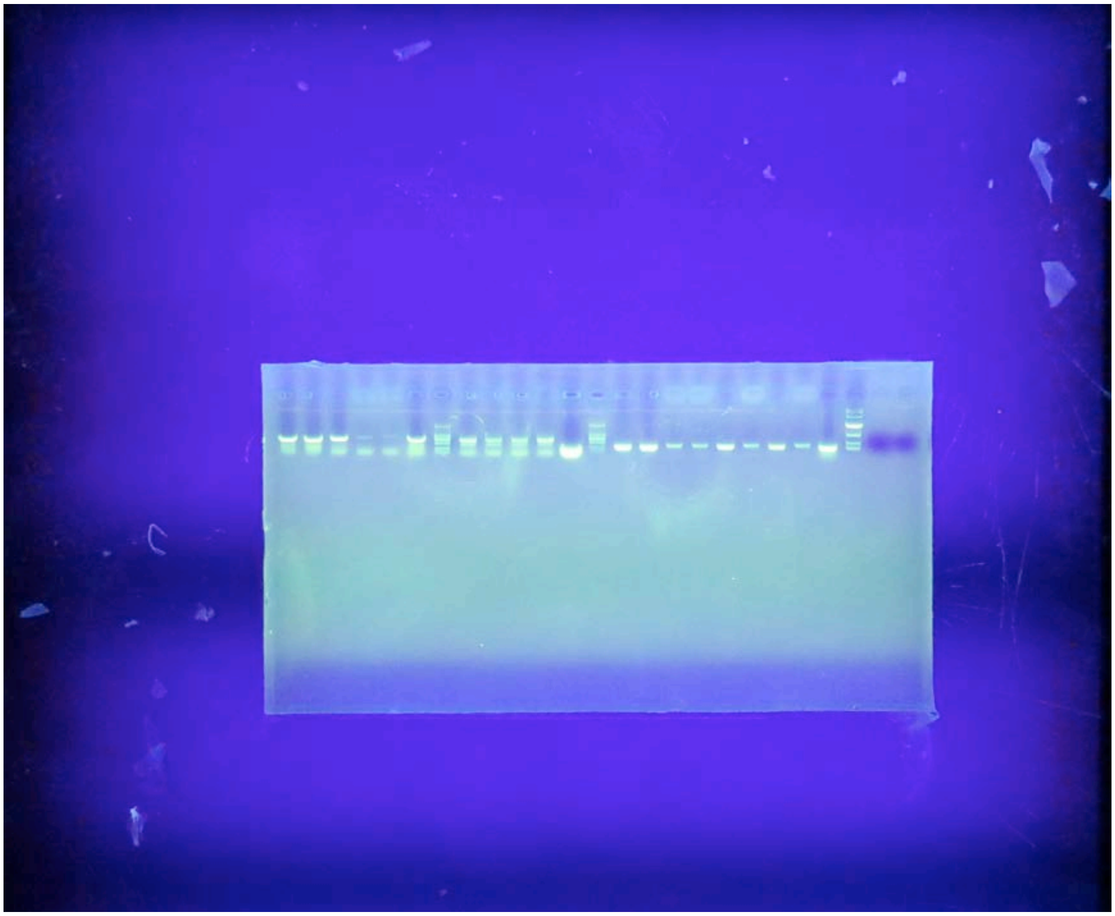

Uncropped gel of supplementary figure 13a
